# Major Quantitative Trait Loci and Putative Candidate Genes for Powdery Mildew Resistance and Fruit-Related Traits Revealed by an Intraspecific Genetic Map for Watermelon (*Citrullus lanatus* var. *lanatus*)

**DOI:** 10.1371/journal.pone.0145665

**Published:** 2015-12-23

**Authors:** Kwang-Hwan Kim, Ji-Hyun Hwang, Dong-Yeup Han, Minkyu Park, Seungill Kim, Doil Choi, Yongjae Kim, Gung Pyo Lee, Sun-Tae Kim, Young-Hoon Park

**Affiliations:** 1 Department of Horticultural Bioscience, Pusan National University, Miryang, Republic of Korea; 2 Department of Plant Science, Seoul National University, Seoul, Republic of Korea; 3 Department of Genetics, University of Georgia, Athens, Georgia, United States of America; 4 Partner Seed, Ansung, Republic of Korea; 5 Department of Integrative Plant Science, College of Biotechnology and Natural Resources, Chung-Ang University, Ansung, Republic of Korea; 6 Department of Plant Bioscience, Pusan National University, Miryang, Republic of Korea; 7 Life and Industry Convergence Research Institute, Pusan National University, Miryang, Republic Korea; Chungnam National University, REPUBLIC OF KOREA

## Abstract

An intraspecific genetic map for watermelon was constructed using an F_2_ population derived from ‘Arka Manik’ × ‘TS34’ and transcript sequence variants and quantitative trait loci (QTL) for resistance to powdery mildew (PMR), seed size (SS), and fruit shape (FS) were analyzed. The map consists of 14 linkage groups (LGs) defined by 174 cleaved amplified polymorphic sequences (CAPS), 2 derived-cleaved amplified polymorphic sequence markers, 20 sequence-characterized amplified regions, and 8 expressed sequence tag-simple sequence repeat markers spanning 1,404.3 cM, with a mean marker interval of 6.9 cM and an average of 14.6 markers per LG. Genetic inheritance and QTL analyses indicated that each of the PMR, SS, and FS traits is controlled by an incompletely dominant effect of major QTLs designated as *pmr2*.*1*, *ss2*.*1*, and *fsi3*.*1*, respectively. The *pmr2*.*1*, detected on chromosome 2 (Chr02), explained 80.0% of the phenotypic variation (LOD = 30.76). This QTL was flanked by two CAPS markers, wsb2-24 (4.00 cM) and wsb2-39 (13.97 cM). The *ss2*.*1*, located close to *pmr2*.*1* and CAPS marker wsb2-13 (1.00 cM) on Chr02, explained 92.3% of the phenotypic variation (LOD = 68.78). The *fsi3*.*1*, detected on Chr03, explained 79.7% of the phenotypic variation (LOD = 31.37) and was flanked by two CAPS, wsb3-24 (1.91 cM) and wsb3-9 (7.00 cM). Candidate gene-based CAPS markers were developed from the disease resistance and fruit shape gene homologs located on Chr.02 and Chr03 and were mapped on the intraspecific map. Colocalization of these markers with the major QTLs indicated that watermelon orthologs of a nucleotide-binding site-leucine-rich repeat class gene containing an RPW8 domain and a member of SUN containing the IQ67 domain are candidate genes for *pmr2*.*1* and *fsi3*.*1*, respectively. The results presented herein provide useful information for marker-assisted breeding and gene cloning for PMR and fruit-related traits.

## Introduction

Watermelon is a fruit crop of the family *Cucurbitaceae* that is known to originate from Africa and is found in the temperate regions of Africa, central Asia, and the Mediterranean [1,2,3]. Watermelon has 11 gametic chromosomes (2n = 2x = 22) and a genome size of approximately 425 Mb [4,5]. It belongs the xerophytic genus *Citrullus* Schrad. ex Eckl. & Zeyh., which comprises 4 diploid species: an annual, wild or cultivated species, *Citrullus lanatus* (Thunb.) Matsum & Nakai, two perennial wild species, *C*. *colocynthis* (L.) Schrad and *C*. *ecirrhosus* Cogn., and an annual wild species *C*. *rehmii* De Winter [6]. *C*. *lanatus* is divided into subspecies that include the widely cultivated forms of red sweet watermelon, *C*. *lanatus* var. *lanatus*, and a preserving melon type of the ancient cultigens, *C*. *lanatus* var. *citroides* [6].

Breeding programs that employ molecular markers can facilitate efficient introgression of features into elite watermelon cultivars to satisfy seed market demands. Molecular markers that are tightly linked to important traits can be useful for marker-assisted selection (MAS), which facilitates the breeding process by selecting traits based on their marker genotype [7,8]. Several molecular markers have been developed for watermelon via genetic map construction. Due to the narrow genetic background and scarce polymorphism [9,10] in *C*. *lanatus* var. *lanatus*, construction of early genetic maps was based on interspecific crosses between *C*. *lanatus* var. *lanatus* and var. *citroides* [4,9,10,11,12]. With the advent of next generation sequencing (NGS), a reference genome sequence for watermelon has become publicly available (Cucurbit Genome Database, http://www.icugi.org/cgi-bin/ICuGI/genome), and resequencing of the whole genomes of diverse watermelon cultivars, including related species, has been reported [5]. NGS of the entire genome and transcriptome facilitated the discovery of a large set of sequence variants such as single nucleotide polymorphisms (SNPs), and enabled the construction of high-resolution intraspecific genetic maps [13] and quantitative trait loci (QTL) analysis [14] in watermelon. However, despite the progress in genomics, publicly available molecular markers for disease resistance and important horticultural traits [14] are very limited when compared to other crop species for which a reference genome sequence is available.

Cloning of disease resistance genes (R genes) from several model plant species showed that a large portion of these R genes encode nucleotide-binding site-leucine-rich repeat (NBS-LRR) proteins [15]. In dicots, NBS-LRR proteins are generally classified into 2 subgroups. One subgroup possesses a domain with significant homology to the Toll/interleukin-1 receptor (TIR) region, whereas the other subgroup is defined by the presence of an N-terminal region containing a coiled-coil (CC) motif [16]. Powdery mildew resistance (PMR) conferred by NBS-LRR genes has been studied in several different plant species. The *Arabidopsis thaliana* locus RESISTANCE TO POWDERY MILDEW 8 (RPW8) contains 2 polymorphic, dominant R genes, *RPW8*.*1* and *RPW8*.*2*, which individually control resistance to a broad range of PM pathogens [17,18]. In melon, a cluster of NBS-LRR genes harbors candidate genes for resistance to different races of PM and other diseases [19,20]. In wheat and barley, non-orthologous genomic regions contain genes homologous to resistance gene analog (RGA)-like NBS-LRR genes, and several QTLs for PMR have been identified and used for map-based cloning of PMR genes [21,22]. These findings indicate that QTLs for PMR in watermelon may also be under genetic control of NBS-LRR class R gene(s).

Genes underlying fruit-shape QTLs have been identified and cloned in tomato (*Solanum lycopersicum* L.), which shows extensive fruit morphological diversity. Variation in tomato fruit shape (FS) is largely contributed to mutations in 4 genes; SUN and OVATE, regulating fruit elongation, and LOCULE NUMBER (LC) and FASCIATED (FAS), regulating locule number and flat fruit shape [23]. SUN is encoded by a member of the IQ domain family, OVATE by a member of the Ovate Family Proteins family, LC by a member of the WOX family, and FAS by a member of the YABBY family [23]. Another plant species showing high diversity in FS is melon, which belongs to cucurbit family along with watermelon. On the basis of genetic linkage maps for melon, a number of QTLs controlling fruit morphology including shape and size has been identified [24]; however, no candidate genes underlying these QTLs have been cloned or functionally characterized. Instead, candidate genes in melon have been suggested based on their homology with gene families of cloned tomato QTLs for fruit morphology [24]. Melon QTLs for fruit morphology were anchored on the melon draft genome sequence, and melon family members of 6 cloned tomato QTLs were identified. The melon QTLs and candidate genes colocalized on the pseudochromosomes, implying that the variation in fruit morphology in different taxa might be controlled by genes of certain ancestral gene families [24].

Here, we report the identification of major QTLs for PMR, seed size (SS; weight), and FS in watermelon, as well as the development of expressed sequence tag (EST) makers linked to those QTLs. In addition, based on R gene analogs and fruit morphology QTLs cloned in tomato, we searched for candidate orthologs for PMR and FS in the watermelon genome sequence and mapped these to the QTLs identified in this study. Therefore, we constructed an intraspecific genetic map based on transcript and genetic variants revealed by using NGS. The results provide useful information that is practically applicable to breeding programs aimed at developing watermelon cultivars with PMR and novel fruit-related traits.

## Materials and Methods

### Plant materials

A crimson-type open-pollinated cultivar ‘Arka Manik’ (AM) from India and a Jubilee-type inbred line ‘TS34’ (TS) developed in Korea were used to develop EST markers and for genetic linkage map construction. F_1_ plants were produced by crossing AM as the maternal parent with TS as the pollen donor. The F_1_ plants were self-pollinated to produce F_2_ progeny. These F_2_ progeny were subsequently self-pollinated and 133 F_3_ families were obtained. All crosses and progeny production were conducted in the greenhouse at NH Seeds in Ansung, Korea between 2008 and 2010.

### Phenotypic analysis

PMR and fruit-related traits including SS and FS were investigated to analyze genetic inheritance and to develop DNA markers linked to these traits. ‘AM’, ‘TS’, F_1_, and F_2_ plants were phenotyped for SS and FS traits, and for PMR, ‘AM’, ‘TS’, F_1_, and F_3_ families were evaluated.

#### PMR

A PM disease assay was conducted in a greenhouse at Pusan National University in Miryang, Korea. Race identification of PM (*Podosphaera xanthii*) inoculum was conducted using 4 melon cultivars (‘Topmark’, ‘PMR1’, ‘PMR6’, and ‘PMR45’) (8 plants per cultivar) as differential hosts. Upon evaluation of the F_3_ progeny, a completely randomized block design was used. Three blocks were tested and 10 plants for each parental line and F_3_ family were included in each block. Seeds were sown in 50-cell trays containing commercial soil mixture (Chungjung Bio; Dongbu Farm Hannong, Seoul, Korea). Seedlings were grown under greenhouse conditions with a daytime temperature of 25–30°C and a nighttime temperature of 15–20°C under > 50% humidity. Preparation of PM inocula and inoculation were conducted as described previously [25]. Disease severity was assessed using 3 rating classes depending on the level of sporulation as described in [25]: 1 (resistance, R) = the resistance level of ‘AM’, no disease symptoms at seedling stage; 2 (intermediate resistance, IR) = the resistance level of F_1_, few colonies present, moderate level of sporulation; 3 (susceptibility, S) = the susceptibility level of ‘TS’, highly profuse sporulation over the entire plant. From these scores, a disease severity index (DSI) was calculated for each cultivar.

#### Fruit-related traits

Phenotyping for fruit-related traits was conducted at the watermelon breeding field of Partner Seed Company (Ansung, Korea) in Konkan, Thailand. The field studies did not involve endangered or protected species. Five ‘AM’, ‘TS’, and F_1_ pants and 133 F_2_ plants were grown and a single fruit was set from each individual plant. Matured watermelon fruits were harvested and their FS and SS were investigated. For FS analysis, each fruit was cut from top to bottom and the width and length of the sections were measured using a graduated ruler. The FS was represented as the FS index (FSI) defined by the ratio of the width to the length. For SS, matured seeds from a harvested fruit were collected, and the weight of 20 seeds (20sw) per individual was measured using an electronic scale.

### RNA sequencing and whole-genome resequencing

#### Transcriptome sequencing and detection of sequence variations

Total RNA was extracted from ‘AM’ and ‘TS’ seedlings at the one-leaf stage. Three seedlings per line were pooled and ground after freezing using liquid nitrogen. The total RNA was purified using a Qiagen RNA purification kit (Qiagen, Hilden, Germany), after which cDNA was synthesized using the Transcriptor First Strand cDNA Synthesis Kit (Roche, Germany) according to the manufacturer’s instructions. The cDNA was sequenced using an Illumina/Solexa Hiseq2000 NGS instrument at the National Instrumentation Center for Environmental management (NICEM) in Seoul National University, Seoul, Korea.

Prior to *de novo* assembly, data preprocessing was performed for each transcriptome library using an in-house preprocessing pipeline [26,27]. First, putative prokaryotic sequences were removed via reference mapping with Bowtie2 [28] using a released bacterial genome as the backbone. Then, duplicated raw sequences resulting from PCR amplification during sequencing were eliminated using an in-house Perl script and low-quality bases were trimmed with CLC NGS Cell (CLC bio, Denmark) with cut-off values Q20. Finally, rRNA sequences were filtered out using SortMeRNA [29].

*De novo* assembly was performed using CLC NGS Cell to construct unigene sets for ‘AM’ and ‘TS’. Orthologous unigene sets were searched and aligned using nucleotide BLAST searches. From the alignments, sequence variations including SNPs, simple sequence repeats (SSRs), and insertions/deletions (indels) were identified.

#### Whole-genome resequencing and comparative SNP analysis

Genomic DNA was extracted from ‘AM’ and ‘TS’ parental lines used for QTL analysis following the method described in [30]. A genomic DNA library with insert sizes of 300~500 bp was constructed from each sample using the TruSeq DNA Sample Preparation Kit (Illumina, San Diego, CA, USA) following the manufacturer’s protocols. Raw sequence data more than 20-fold of the watermelon reference genome size (425 Mb) were obtained from each genomic library by paired-end sequencing (2 × 100 bp) using a HiSeq 2000 (Illumina) platform. Watermelon genome (97103) version 1 with annotations available from the Cucurbit Genomics Database (CuGenDB) (http://www.icugi.org/cgi-bin/ICuGI/index.cgi) was used as a reference for assembly.

The raw sequence reads from each sample were pre-processed using the CLC Genomics Workbench software version 7.0.3 (CLC bio). First, adaptor sequences were trimmed off the raw reads. Then, low-quality bases (< Q20) were marked as “N.” Sequences with less than 90 base pairs or with “N”s more than 10% of their total read length were discarded. The quality-trimmed sequence reads were aligned to the watermelon reference genome using the CLC Genomics Workbench with the following parameter settings: minimum contig size = 200; mismatch cost = 2; gap cost = 3; deletion cost = 3; identity (similarity) = 80%; HSP coverage (length fraction) = 50%. After read mapping, a consensus sequence was extracted for each sample and annotations were transferred from the reference genome for functional analysis.

SNPs were identified using the Probabilistic Variant Caller program in the CLC Genomics Workbench with the following parameters: minimum coverage = 5; variant probability ≥ 90%. The algorithm combines a Bayesian model and a Maximum Likelihood approach to calculate prior and error probabilities for the Bayesian model. Non-synonymous SNPs, which cause amino acid changes in the coding region, were filtered out using the Amino Acid Change tool in the CLC Genomics Workbench. To compare the SNPs between samples, the Convert to Track tool was used to view the distribution of variants on each chromosome. To identify sample-specific SNPs, we used the Compare Sample Variants tool.

### Development of EST and gene-based markers for genetic mapping

CAPS markers were developed based on SNPs located in restriction enzyme recognition sites using the SGN CAPS Designer tool (http://solgenomics.net/tools/caps_designer) and Primer3 (http://bioinfo.ut.ee/primer3-0.4.0/). For SNPs that did not create a restriction enzyme recognition site, derived-CAPS (dCAPS) markers were developed using dCAPS Finder 2.0 (http://helix.wustl.edu/dcaps/dcaps.html). Sequence characterized amplified region (SCAR) markers were developed by designing primer sets flanking indel regions (>5 bp) in unigene sequences using Primer3. The primers (synthesized by Bioneer, Daejoen, Korea) were evaluated for their ability to detect polymorphism between ‘AM’ and ‘TS’. Genomic DNA extraction, cloning, PCR, enzyme restriction, and agarose gel electrophoresis used for marker development were conducted as described previously [31]. In addition to the newly developed primers, watermelon EST-SSR primers previously reported in [32] were used to screen for polymorphisms between ‘AM’ and ‘TS’ and used for genetic map construction.

NBS-LRR gene homologs in watermelon were retrieved from the draft watermelon genome sequence (97103, version 1) deposited to the CGD. Candidate fruit morphology genes were identified using BLASTP searches with tomato SUN, OVATE, LOCULE NUMBER, and FASCIATED (FAS) protein sequences achieved from the Sol Genome Network (SGN) (http://solgenomics.net/, ITAG2.3 release) against the melon reference genome sequence at Melonomics (https://melonomics.net/, v3.5). The melon genes retrieved were blasted to watermelon genome sequence (97103) to identify gene members of the family, and the locations of these genes were anchored on the genetic map.

### Genetic map construction and QTL analysis

For genetic map construction, genotyping of ‘AM’, ‘TS’, and the F_2_ population using CAPS, dCAPS, SCAR, and EST-SSR markers was performed based on touchdown PCR and agarose gel electrophoresis as described previously [31]. Only polymorphic codominant markers segregating in a predicted Mendelian F_2_ ratio (1:2:1) as confirmed by chi-square (*X* ^*2*^) analysis employing JoinMap Version 4.0 software [33] were included for genetic linkage analysis. Genetic linkage maps were constructed using the JoinMap Version 4.0 software, with the Kosambi mapping function [34]. Logarithm of odds (LOD) scores from 3.0 to 4.0 and a maximum genetic distance of 45.0 cM were taken as thresholds for the determination of linkage groups (LGs) and genetic distance (GD; cM). Linkage groups were named according to the corresponding chromosome following Ren et al. [4]. The genetic map order of markers on each linkage group was confirmed by comparison with their physical locations on the reference watermelon genome sequence (97103). QTL analysis and mapping were initially carried out by interval mapping (IM) to identify genomic regions responsible for the traits. Subsequently, composite interval mapping (CIM) was conducted using the multiple QTL mapping (MQM) function of the MapQTL software v5.0 [35]. The genome-wide LOD threshold at the 5% significance level was determined by 1000 permutation tests for each trait. The phenotypic variation explained by each locus was calculated from the value at the QTL peaks as indicated by IM and CIM.

## Results

### EST-based genetic map construction

#### Transcriptome analysis

Datasets of 3.64 Gb (total number of reads: 36,036,775) and 2.96 Gb (29,262,424 reads) were generated by NGS of total RNA from ‘AM’ and ‘TS’, respectively. *De novo* assembly resulted in 27,836 and 29,079 contigs with average lengths of 1,054 bp and 1,037 bp for ‘AM’ and ‘TS’, respectively. Sequence comparisons of the homologous contigs between ‘AM’ and ‘TS’ yielded 14,910 sequence variants, 7,455 of which were highly reliable SNPs and indels that were considered for EST marker development. These 7,455 SNPs and indels were detected from 2,819 unigenes and their locations on the watermelon pseudomolecule (Cucurbit Genome Database, http://www.icugi.org/cgi-bin/ICuGI/genome) are depicted in S1 File.

#### Development of EST-based markers

We selected 522 unigenes that contained sequence polymorphisms (SNPs or indels) between ‘AM’ and ‘TS’ and were evenly distributed over the 11 chromosomes. PCR primer pairs flanking the polymorphisms were designed to develop PCR-based codominant markers. Primer pairs for 455 CAPS and 11 dCAPS were designed based on SNPs, while those for 56 SCARs were generated based on indels. In addition to these markers, 54 watermelon EST-SSRs previously reported by Hwang et al. [32] were included in the PCR evaluation of the markers’ ability to detect polymorphism between ‘AM’ and ‘TS’. Of the 576 markers, 312 (54.16%) (229 CAPS [50.32%] 4 dCAPS [36.36%], 29 SCAR [51.78%], and 50 EST-SSR [92.59%]) produced distinct polymorphic banding patterns for the two genotypes. Among these, 97 markers that showed dominant patterns or were ambiguously scored were excluded, and a final set of 205 markers (S1 Table) was used for genotyping of the F_2_ population and genetic linkage map construction.

#### Genetic linkage map construction

**Fig 1:**
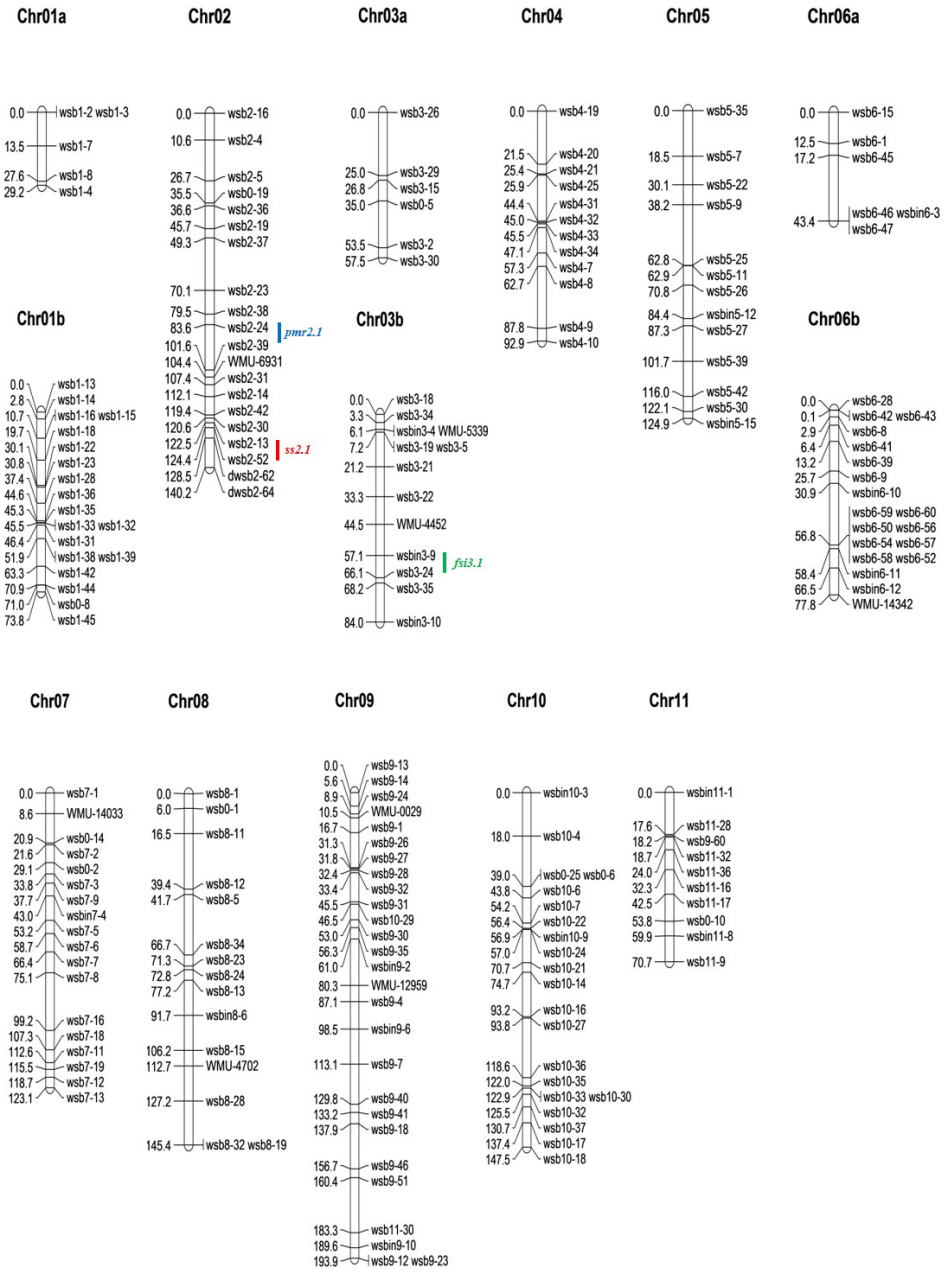
An intraspecific genetic map for watermelon constructed using an F_2_ population from the cross ‘Arka Manik’ × ‘TS34’ and the location of 3 major QTLs for powdery mildew resistance (PMR), seed size (SS), and fruit shape index (FSI) identified by interval mapping (IM). Chromosomes 1 to 11 are according to [4]. Numbers on the left side correspond to the distance in cM from the top of each chromosome. QTL positions were defined by the interval of the strongest linked markers flanking the QTL. Major QTL for PMR, SS, and FSI are represented by the blue, red, and green color, respectively.

An intraspecific watermelon genetic linkage map was constructed using 133 F_2_ plants segregating for PMR, SS (represented by 20sw), and FSI, and 204 DNA markers (Fig 1). Four of the 205 markers were eliminated during mapping because of segregation distortion from Mendelian expectation (*P* < 0.05), and 1 unlinked marker is not reported herein. Overall, 14 LGs were defined by 204 markers including 174 CAPS, 2 dCAPS, 19 SCAR, and 8 EST-SSR markers. This unsaturated melon map spanned 1,404.3 cM, with a mean marker interval of 6.9 cM and an average of 14.6 markers per LG (Table 1). Marker order was, with rare exceptions, consistent with the watermelon physical map in the CuGenDB.

**Table 1:** Distribution of molecular markers and major QTLs among 14 linkage groups constructed using an F_2_ population from the cross ‘Arka Manik’ and ‘TS34’.

LG	Map length (cM)	No. of markers	Marker density (cM/marker)	Marker types			QTL
				CAPS	SCAR	SSR	
Chr. 1a	29.2	5	5.84	5	-	-	-
Chr. 1b	73.8	19	3.88	19	-	-	-
Chr. 2	140.2	20	7.01	19	-	1	*pmr2*.*1*, *ss2*.*1*
Chr. 3a	57.5	6	9.58	6	-		-
Chr. 3b	84.0	13	6.46	8	3	2	*fsi3*.*1*
Chr. 4	92.9	12	7.74	12	-	-	-
Chr. 5	124.9	13	9.60	11	2	-	-
Chr. 6a	43.4	6	7.23	5	1	-	-
Chr. 6b	77.8	19	4.09	15	3	1	-
Chr. 7	123.1	18	6.84	15	2	1	-
Chr. 8	145.4	15	9.69	13	1	1	-
Chr. 9	193.9	27	7.18	22	3	2	-
Chr. 10	147.5	21	7.02	18	3	-	-
Chr. 11	70.7	10	7.07	8	2	-	-

### Phenotypic evaluation and QTL analysis

#### PMR evaluation

**Fig 2:**
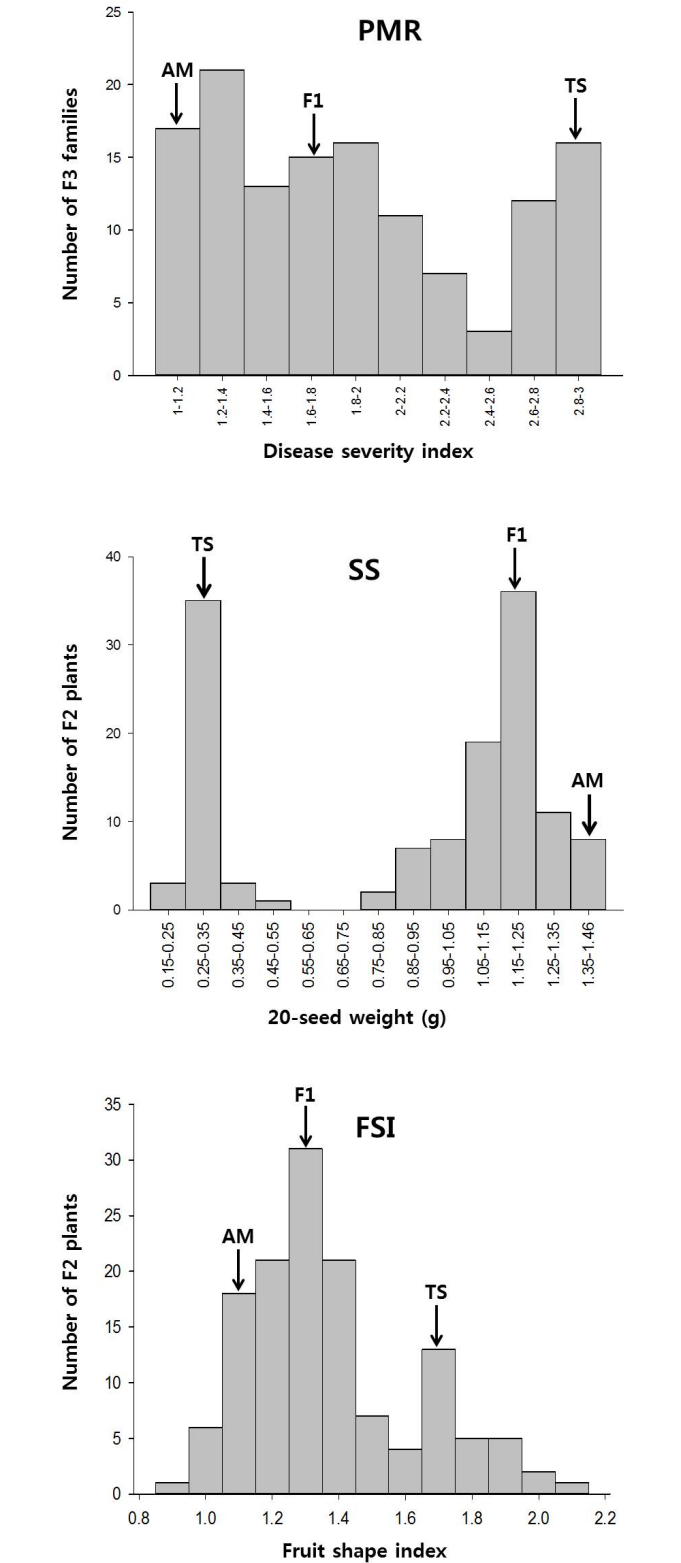
PMR phenotypic distribution of F_2_ progeny from the cross ‘Arka Manik’ × ‘TS34’.

Identification of the PM race using differential hosts of melon indicated that the inoculated race was 1W, as reported previously [25]. Among ‘PMR1’, ‘PMR6’, ‘PMR45’, and ‘Topmark’, ‘Topmark’ was susceptible, while other cultivars did not show any disease symptoms after inoculation, indicating that the inocula had the same pathogenic reaction as race 1W [37,38]. The DSI showed that ‘AM’ was highly resistant (DSI = 1.0), while ‘TS’ was susceptible (DSI = 3.0) to race 1W. Additionally, 131 F_3_ families segregated with the DSI ranging from 1 to 3, while seeds of two F_3_ families failed to germinate and could not be evaluated for PMR. The phenotypic distribution of the F_3_ families implied that PMR in ‘AM’ is under simple genetic control, and possibly subject to an incompletely dominant effect by a major gene (Fig 2). QTL analysis based on the constructed genetic linkage map revealed a major locus (named *pmr2*.*1* in this study) for PMR located on Chr02 (Table 2, Fig 1). The major QTL *pmr2*.*1* could explain 80.0% of the phenotypic variation (LOD = 30.76). Two CAPS markers, wsb2-39 (13.97 cM, SNP location 27,472,123–27,473,080 bp) and wsb2-24 (4.00 cM, SNP location 25,214,171–25,214,903 bp), are tightly linked with *pmr2*.*1* (Table 3). Only one putative minor QTL for PMR explaining 4.9% phenotypic variation (LOD = 5.12) was detected between the two CAPS markers wsb2-13 and wsb2-52 on Chr02; however, no QTL was detected in other genomic regions.

**Table 2:** QTL mapping results for PMR, SS, and FSI in the F_2_ population from the cross ‘Arka Manik’ × ‘TS34’.

Trait	Marker/QTL	Chromosome	Position (cM)	LOD	Variance	R^2^ (%)	Additive	Dominance
PMR	wsb2-24		83.582	25.24	0.111982	66.9	-0.650317	-0.227023
	*pmr2*.*1*	2	87.582	30.76	0.0676163	80.0	-0.722705	-0.259516
	wsb2-39		101.552	13.38	0.183179	45.9	-0.602451	-0.224135
SS	wsb2-52		124.441	51.98	0.0282078	83.7	0.448240	0.362165
	*ss2*.*1*	2	123.504	68.78	0.0133388	92.3	0.447931	0.398613
	wsb2-13		122.504	57.14	0.0235608	86.4	0.448384	0.372659
FSI	wsbin3-9		57.140	18.76	0.0259392	59.7	-0.252392	-0.154192
	*fsi3*.*1*	3	64.140	31.37	0.0130317	79.7	-0.322514	-0.133143
	wsb3-24		66.054	24.59	0.0198845	69.1	-0.295196	-0.116682

**Table 3:** Primers for the major QTL-flanking markers shown in Table 2.

QTL	Flanking Marker	Marker type^a^	Primer		EST location	Enzyme
			Forward(5’-3’)	Reverse (5’-3’)	Chromosome (bp)	
*pmr2*.*1*	wsb2-24	CAPS	ATGGTTTACGGCAGAGCAAT	GGAGGAATCGAAGAACTCCA	2(25214171–25214903)	*Bsl*I
	wsb2-39	CAPS	TTTTGCGACGATTTTCTTCC	GTAGCGGCGATGAACAGAGT	2(27472123–27473080)	*Bcl*I
*fsi3*.*1*	wsbin3-9	SCAR	CGCTGGCTACAAGTTCACAA	GTGATCAGGTCTGGGCAGTT	3(26050670–26055200)	-
	wsb3-24	CAPS	CCATAATCCGATCCAATGCT	GGAAAGGGATGGGTGAAAGT	3(26945390–26945800)	*Hinc*II
*ss2*.*1*	wsb2-13	CAPS	CAAATCGAAGGTTGAAAATGC	TGCCCTTGTAGACTCCCTTG	2(29842335–29843159)	*Taq*I
	wsb2-52	CAPS	TGATGGTCCATGGCTACTGA	ACTTGGCAAACCTCGTGAAG	2(29993416–29992300)	*HinfI*

^a^ CAPS, cleaved amplified polymorphic sequence; SCAR, sequence-characterized amplified region; SSR, simple sequence repeat.

#### FS evaluation

The FS of watermelon is generally classified as spherical, oval, or elongate (UPOV, http://www.upov.int). The FSI is calculated as the ratio of the diameter to the length. The FSI of ‘AM’ and ‘TS’ was 1.1 and 1.7, which fall into spherical and elongate type, respectively (UPOV, http://www.upov.int). The FSI range of the F_2_ population was 0.9–2.1. The phenotypic distribution of 133 F_2_ plants indicated that watermelon FS is controlled by a major locus in an incompletely dominant manner (Fig 2). QTL mapping revealed that this major QTL (named *fsi3*.*1* in this study) was located on Chr03, and explained 79.7% of the phenotypic variation (LOD = 31.37) (Table 2, Fig 1). The major QTL *fsi3*.*1* is flanked by the closely linked CAPS markers wsb3-9 (7.00 cM, SNP location 26,050,670–26,055,200 bp) and wsb3-24 (1.91 cM, SNP location 26,945,390–26,945,800 bp) (Table 3). No QTL was detected in other genomic regions.

#### SS evaluation

The 20sw of ‘AM’ and ‘TS’ was 1.39 g (normal seed size) and 0.32 g (tomato seed size), respectively. The phenotypic distribution of the 20sw in 133 F_3_ families indicated that SS is controlled by a single dominant gene (Fig 2). Specifically, the 20sw of 45 F_3_ families ranged from 0.16 g to 0.47 g, while that of 80 F_3_ families ranged from 1 g to 1.46 g. The 10 remaining F_3_ families showed an intermediate sw20 ranging from 0.77 g to 0.97 g, possibly due to environmental effects. QTL analysis based on the constructed genetic linkage map revealed a major locus (named *ss2*.*1* in this study) for SS (20sw) located on Chr02 (Table 2, Fig 1). The major QTL *ss2*.*1* explained 92.3% of the phenotypic variation (LOD = 68.78) and was located close to the PMR QTL *pmr2*.*1*. The QTL *ss2*.*1* is tightly linked with CAPS markers wsb2-13 (1.00 cM, SNP location 29,842,335–29,843,159 bp) and wsb2-52 (0.94 cM, SNP location 29,993,416–29,992,300 bp) (Table 3). One additional QTL was detected between the two CAPS markers wsb2-24 and wsb2-39 on Chr02; however, it explained only 6.4% phenotypic variation (LOD = 4.91).

### Mapping of putative candidate genes

#### Whole-genome resequencing

NGS-based whole-genome resequencing was employed to detect genetic variation between the parental lines and to develop SNP markers for QTL analysis. The results indicated that the NBS-LRR and SUN genes are good candidates for PMR and FS, respectively, in watermelon. Resequencing of ‘AM’ and ‘TS’ generated 13.1 and 19.4 million paired-end reads, respectively, with and average read length of 101 bp. Pre-processing of the raw sequences resulted in 11.8 and 17.9 million reads that accounted for total lengths of 1.08 Gb and 1.62 Gb, with approximately 3.34× and 4.57× genome coverage for ‘AM’ and ‘TS’, respectively. Trimmed sequence reads were aligned to the reference genome 97103 (total chromosome length = 355.24 Mb), and 91.87% and 89.41% of the reads were mapped, covering 89.36% and 89.41% of the reference genome, for ‘AM’ and ‘TS’, respectively. From the comparison with the reference genome (read depth ≥ 90%), we identified 199,189 homozygous SNPs and 13,276 homozygous indels for ‘AM’ and 153,286 homozygous SNPs and 9,981 homozygous indels for ‘TS’. Between ‘AM’ and ‘TS’, a total of 164,438 SNPs and 11,235 indels were detected.

#### Putative candidate gene search

In total, 87 R genes including NBS-LRR class genes were found by searching the CuGenDB. Among these genes, 16, 25, and 10 genes belonged to the CC-NBS, TIR-NBS, and NBS-LRR families, respectively. Most of the remaining genes were annotated as disease resistance-responsive genes (S2 Table). The R genes were found to be located throughout the watermelon genome. For instance, all 11 psuedochromosomes of the reference genome contained at least 1 R-like gene, while Chr02, Chr08, and Chr10 carried the highest numbers of R genes (19, 14, and 10, respectively) (S2 Table). The R genes tended to cluster at specific genomic regions of each chromosome, as has been frequently observed for R genes in various other plant species [15]. A major QTL for PMR *pmr2*.*1* was detected on Chr02 and was flanked by two CAPS markers wsb2-24 and wsb2-35. Proximal to this map interval, a cluster of 8 R genes on Chr02 (Cla019831–Cla019863 in S2 Table) was identified and we presumed that R genes in this cluster are putative candidate genes for PMR to race 1W.

BLASTing of tomato fruit morphology gene families revealed highly homologous sequences to SUN, OFP, WOX, and YABBY gene families in watermelon. A total of 24 genes showing a highest hit (E-126 ~ 0) to protein sequences of 24 melon SUN gene members (designated as “CmSUN” in [24]) were selected (S3 Table). These SUN gene homologs in watermelon (designated as “ClSUN” in this study) were scattered over most of the chromosomes except Chr02 and Chr11. In the genetic map, two CAPS markers flanking the *fsi3*.*1* QTL were located at 26.0 Mb and 26.9 Mb of Chr03. We identified a watermelon SUN gene member [ClSUN-8 (Cla011257), 26846490 bp, homologous to CmSUN-14 (E-160)] that was located in this physical map interval and is a candidate *fsi3*.*1* gene.

#### Sequence variant detection for putative candidate genes

Resequencing data of ‘AM’ and ‘TS’ were compared to identify the sequence variants in putative candidate genes for *pmr2*.*1* and *fsi3*.*1* (Table 4). Of 8 NBS-LRR genes located on Chr02, only 3 showed sequence variance between the 2 parents. All these variants were SNPs; no indel was detected. Cla019831 had 2 SNPs in exon 2, Cla019844 harbored 2 SNPs in exons 1 and 3, and Cla019855 had 5 SNPs in exon 4. Of 3 FS-related genes located on Chr03, 2 SUN genes showed SNPs between both parents, but no indels. Cla011257 had 3 SNPs in exons 2 and 4, and Cla001773 harbored 3 SNPs in exons 1 and 2. Amino acid sequence changes were found for 3 SNPs. These SNPs were used to develop allele-specific codominant PCR-based markers (Table 5). To develop CAPS, SNPs were screened for restriction enzyme recognition sites. For Cla19831, A/G located at 26751854 bp was located in a *Taq*I recognition site. The SNPs for Cla019844 (T/A) and Cla011257 (G/A) were not recognized by any restriction enzyme; thus, dCAPS markers were designed for these SNPs (Table 5). PCR and enzyme digestion yielded PCR fragments of expected size for all CAPS and dCAPS markers.

**Table 4:** R genes and FS-related genes detected from chromosomes with PMR and FSI major QTLs, and SNPs in these gene between ‘Arka Manik’ and ‘TS34’. The genes and SNPs for which PCR markers were developed are underlined.

Trait	Gene	Gene ID	Location		Gene function annotation	SNP(AM/TS)	
			Chr.	Reference genome(bp)		Nucleotide	Position (bp)
PMR	R-gene	Cla019831	2	26750001–26753327	Disease resistance protein (IPR002182 NB-ARC)	A/G	26751854
						A/C	26752052
		Cla019844	2	26582380–26589679	Cc-nbs-lrr resistance protein (IPR002182 NB-ARC)	C/T	26583608
						C/T	26583793
						T/G	26584613
						T/A	26586794
		Cla019850	2	26501165–26506002	Resistance protein RGC2 (Fragment) (IPR001611 Leucine-rich repeat)	-	-
		Cla019854	2	26456943–26459976	TIR-NBS disease resistance-like protein (IPR000157 Toll-Interleukin receptor)	-	-
		Cla019855	2	26449200–26453033	TIR-NBS disease resistance protein (IPR000157 Toll-Interleukin receptor)	T/C	26449686
		Cla019856	2	26439873–26444126	TIR-NBS disease resistance-like protein (IPR000157 Toll-Interleukin receptor)	-	-
		Cla019857	2	26432098–26437657	TIR-NBS disease resistance-like protein (IPR000157 Toll-Interleukin receptor)	-	-
		Cla019863	2	26383499–26388744	TIR-NBS disease resistance-like protein (IPR000157 Toll-Interleukin receptor)	-	-
FSI	WOX	Cla008252	3	1542265–1543627	WOX2 (WUSCHEL RELATED HOMEBOX 2); transcription factor	-	-
	SUN	Cla011257	3	26846490–26847637	IQD26 (IQ-domain 26); calmodulin binding	C/T	26846636
						G/A	26846820
						C/T	26847041
		Cla001773	3	21241760–21249007	IQD26 (IQ-domain 26); calmodulin binding	C/A	21243320
						C/T	21243489
						C/T	21245913

**Table 5:** Putative candidate gene-based markers located on major QTL regions for PMR and FS.

Trait	Gene ID	Marker type^a^	Primer sequence (5’– 3’)	SNP location [Chr.(bp)]	Enzyme	Product size (bp)^b^	
						AM	TS
PMR	Cla019831	CAPS	F:CTTTTGCTTGCATTGTGCAT	2(26751854)	*Taq*I	527	334,223
			R:GGATGCAAAGGAGCTGTTTC				
	Cla019844	dCAPS	F:ATTGAAGACCGTCCTGCCTTTCTCATAAAAAGT	2(26586794)	*Mse*I	160	192
			R:GCGCAGCCAAATAATTCAGT				
FSI	Cla011257	dCAPS	F:CCTATTTCACCAAACTCTCTCG	3(26846820)	*EcoR*I	345	373
			R:TCCACTAAGACTACTTCTCGATTCCATGAAT				

^a^CAPS, cleaved amplified polymorphic sequence; and dCAPS, derived-cleaved amplified polymorphic sequence

^b^AM, ‘Arka Manik’; and TS, ‘TS34’

#### QTL analysis for putative candidate gene-based markers

**Fig 3:**
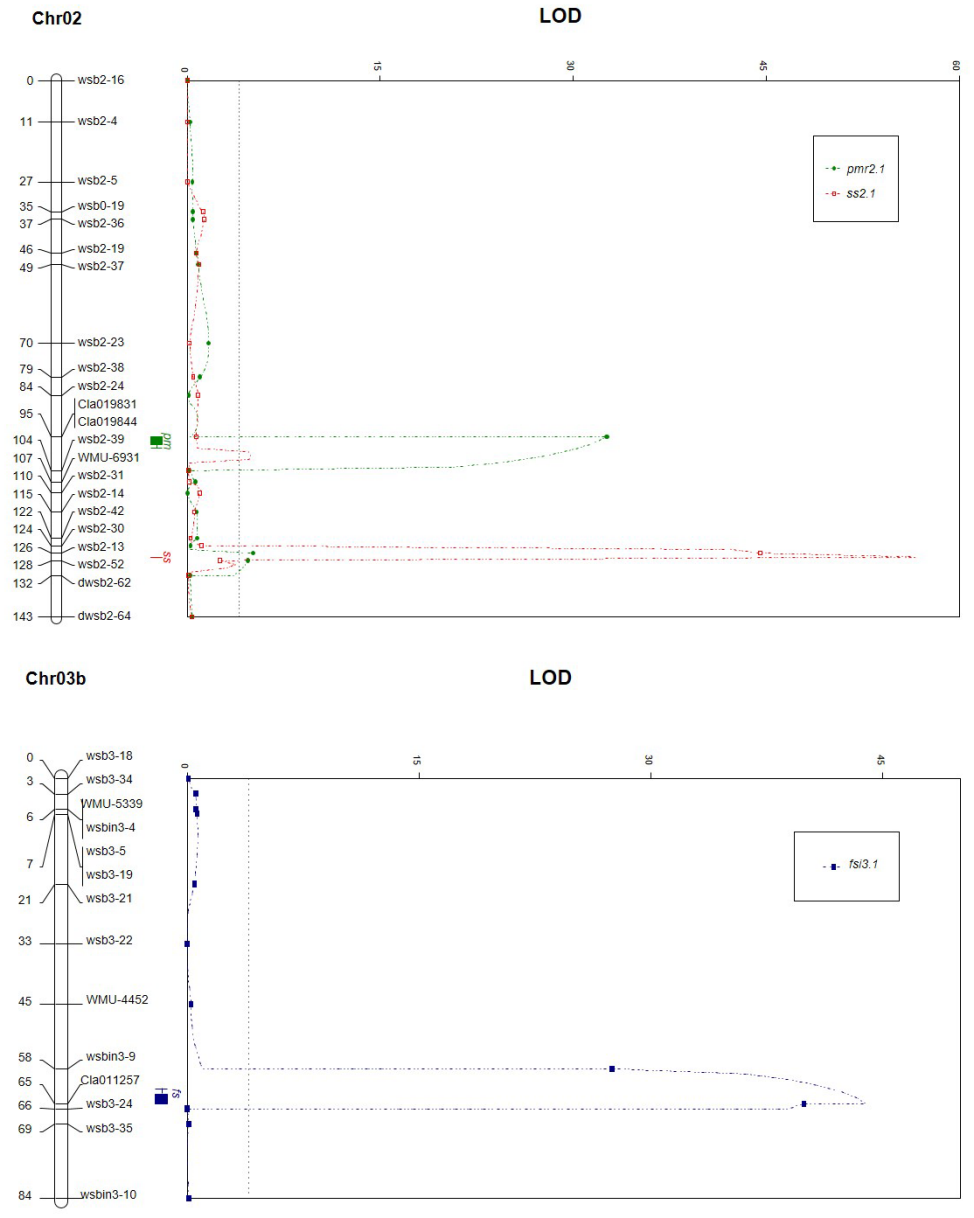
Genetic mapping of two NBS-LRR gene-based markers and a SUN gene homolog-based marker, and composite interval mapping (CIM) of QTLs associated with powdery mildew resistance (PMR), seed size (SS), and fruit shape index (FSI) on Chr02 and Chr03. In QTL LOD graphs, LOD thresholds for 133 F_2_ plants is 4.2.

To analyze colocalization of the putative candidate genes with the major QTLs for PMR and FSI, genetic linkage groups for Chr02 and Chr03 were reconstructed by adding 3 putative candidate gene-based markers. In this reconstructed map (Fig 3), 2 markers for members of the NB-ARC gene family (Cla19831 and Cla19844) cosegregated and were mapped on a genomic region between wsb2-24 and wsb2-39, which were the EST markers flanking the major QTL (*pmr2*.*1*) for PMR in the original map (Fig 1). NB-ARC2.1 and NB-ARC2.2 were approximately 0.2 Mb apart in physical distance, but no recombination occurred in the 133 F_2_ progenies. NB-ARC2.1 and NB-ARC2.2 were mapped at 11.7 cM from wsb2-24 and 8.9 cM from wsb2-39, respectively. For FSI, the dCAPS marker derived from the SUN gene homolog (Cla011257) was mapped at a genomic region between wsbin3-9 and wsb3-24, which flanked the major QTL (*fsi3*.*1*) for FSI. This marker was located 7.8 cM from wsbin3-9 and 1.1 cM from wsb3-24. QTL mapping using CIM was conducted for Chr02 and Chr03, and major QTLs explaining 81.3% for PMR and 74.9% for FSI were detected at 3 putative candidate gene-based markers (Fig 3). Additionally, two putative minor QTLs for PMR and SS that were detected with IM were confirmed by CIM, although their attributions to total phenotypic variation were very small (Fig 3).

## Discussion

From a breeding point of view, diverse fruit-related characteristics including FS, fruit size, rind pattern, fruit flesh color, and SS and seed coat color are important for the global seed market [36,37,38]. Consumers in different countries and geographical regions prefer distinct watermelon traits. For example, Crimson-type seedless cultivars are popular in the United States and Europe, small-sized ice-box-type fruits are popular in Southeast Asia, Jubilee-type round fruits in China, and Jubilee-type broad elliptic fruits in South Korea [34]. Cultivars with high citrullin, β-carotene, and lycopene content are in increased demand, as are those with small or less seeds, diverse flesh colors, and flesh hardness [39].

In addition to fruit-related horticultural traits, disease resistance to diverse pathogens such as viruses, *Fusarium* sp., mildews, and nematodes are important goals for watermelon breeding. PM of cucurbit crops is a fungal disease caused by *P*. *xanthii* (castagne) U. Braun & N. Shishkoff [30]. Two distinct races (1W and 2W) of *P*. *xanthii* pathogenic to watermelon have been identified using a set of differential melon cultivars [40,41]. Identification of the PM race predominant in South Korea and genetic inheritance of PMR in an Indian open-pollinated cultivar ‘Arka Manik’ have been studied previously [25]. PMR is especially important as PM outbreaks are on the rise with increasing greenhouse cultivation and as climate change creates a more subtropical environment in South Korea, which favors PM infection. Generally, PM occurs regardless of cultivation practice or location [42]. Due to the lack of resistant cultivars, the disease is mainly controlled by repeated applications (6–7 times) of fungicides throughout the growing season.

In the present study, we confirmed that the disease response of melon differential hosts was the same for race 1W, as reported previously [25]. QTL analysis indicated that the resistance to 1W in ‘AM’ was controlled by a major QTL with incomplete dominance. These results corroborated those of a previous study on the genetic inheritance of PMR in ‘AM’ that was assessed using F_1_, F_2_, and reciprocal backcross populations derived from a cross with a PM-susceptible pollen donor ‘HS3355’ [25]. In addition, we have reported the development of a SNP marker, MCA-A/G, which was tightly linked to PMR, based on bulked segregant analysis of near-isogenic ‘AM’ lines using random amplified polymorphic DNA [25]. However, no detailed studies on genetic mapping and chromosomal localization of PMR loci in watermelon have been published to date. In our QTL analysis of PMR, a major QTL explaining 80.0% of the phenotypic variation was identified on Chr02 in a region flanked by two CAPS [wsb2.24 (25,214,171–25,214,903 bp) and wsb2-39 (27,472,123–27,473,080 bp)]. A BLAST search of the reference genome using the DNA sequence of the SNP marker MCA-A/G [25] revealed that it was physically located at 26,782,903 bp on Chr02 (data not shown); thus, PMR localized to the same genomic region in two independent studies.

Furthermore, we identified a cluster of 8 NBS-LRR genes proximal to *pmr2*.*1*, and QTL mapping of 2 members of this cluster (Cla019831 and Cla019844 cosegregated in the map) suggested that the cluster of NBS-LRR genes identified on Chr02 harbors putative candidate genes for resistance to race 1W of *P*. *xanthii*. The NBS-LRR genes have been reported to be candidate genes for PMR in several plant species such as *A*. *thaliana* [17,18] and melon [19]. The best BLASTP hits for Cla019831 and Cla019844 were the melon genes XP_008455702.1 and XP_008441731.1, respectively. These putative genes contain two amino acid domains (RPW8 and AAA) characteristic for RPW8 that confers broad-spectrum PMR to Arabidopsis [17] (S2 File). In Arabidopsis, RPW8.1 and RPW8.2 couple the recognition of PM pathogens to induction of the hypersensitive response, which is associated with salicylic acid accumulation and expression of pathogenesis-related genes [18].

FS is known to be controlled by a single, incompletely dominant gene, and F_1_ plants from elongate (*OO*) type × spherical (*oo*) type normally produce fruits with oval shape (*Oo*) [43]. The gene effect of incomplete dominance on FS was also estimated from our study, as indicated by the discrete qualitative segregation pattern shown in Fig 2. Continuous variation may be due to environmental effects on FS. A major QTL for FS on Chr03 has been previously reported in [14] and [44]. Using an elite × elite (KBS × NHM) population, the authors detected 2 minor QTLs controlling FS from Chr02 [*Qfsi2*, R^2^(%) = 2.8, LOD = 3.3], and Chr10 [*Qfsi10*, R^2^(%) = 3.0, LOD = 3.2], as well as a major QTL from Chr03 [*Qfsi3*, R^2^(%) = 56.6, LOD = 30.8]. According to [14], the major QTL *Qfsi3* was flanked by 2 SNP markers, NW0250956 and NW0248107, located at 25,980,598~25,980,718 bp and 26,945,214~26,945,334 bp of the reference genome, respectively. Similarly, we identified a major QTL *fsi3*.*1* explaining 79.7% of the phenotypic variation (LOD = 31.37) located on Chr03, and 2 SNPs of flanking CAPS markers wsb3-9 and wsb3-24 were located at 26,050,670~26,055,200 bp and 26,945,390~26,945,800 bp, implying that *Qfsi3* and *fsi3*.*1* may be the same locus.

Additionally, QTL mapping of a watermelon gene (Cla011257, ClSUN-8) homologous to tomato FS genes suggested that a member of *SUN* may be a putative candidate gene for *fsi3*.*1* controlling FS in watermelon (S3 File). *SUN*, one of the major genes controlling the elongated FS of tomato was found to encode a member of the IQ67 domain-containing family [45,46]. *SUN* controls tomato shape through redistribution of mass that is mediated by increased cell division in the longitudinal and decreased cell division in the transverse direction of the fruit [45]. Genomic location of watermelon members of other fruit morphology gene families like OFP, WOX, and YABBY indicated that these genes are not linked to *fsi3*.*1* and possibly are not directly involved in determining the different FS between ‘AM’ (spherical) and ‘TS’ (elongated).

The genetic inheritance of watermelon SS has been reported in [47,48]. In these studies, diverse crosses were made between watermelon lines with different SS—giant seed (GS), large seed (BS), normal medium seed (NS), small seed (SS), and tomato seed (TS) sizes—and the resulting populations were evaluated for SS inheritance. Based on a cross between GS and TS, the authors speculated that at least 6 quantitative genes were involved in SS, with 3 major genes between NS and TS. Our QTL analysis for NS (‘AM’) and TS (‘TS’) indicated that a major locus on Chr02, *ss2*.*1*, significantly influences SS. Locus *ss2*.*1*, which explained 92.3% of the phenotypic variation, was located in the vicinity of the wsb2-13 marker. In addition, [14] and [49] reported 2 major QTLs for SS based on the elite × elite (KBS × NHM) population, which were detected on Chr06 (*Q100swt6*, R^2^(%) = 26.9, LOD = 13.5) and Chr02 (*Q100swt2-1*, R^2^(%) = 4.8, LOD = 3.1). According to [14], the minor QTL *Q100swt2-1* on Chr02 was flanked by 2 SNP markers, NW0250227 and NW0249226, located at 27,192,980~27,193,071 bp and 28,849,829~28,849,949 bp of the reference genome, respectively. The studies for seed size inheritance [47,48] and QTL mapping [14,49] indicate that *ss2*.*1* and *Q100swt2-1* are members of the multiple QTL determining a wide range of the watermelon seed sizes, and are possibly allelic or closely linked genes.

Interestingly, putative minor QTLs for PMR and SS revealed by CIM were located at the same locations as the major QTL for SS and PMR, respectively (Fig 3). This implies that SS may somehow possitively influence PMR, although its effectiveness is very low. In this study, for ‘TS’ with tomato seed size, a relatively low seed germination rate and slow and weak vegetative growth at seedling stage were observed as compared to ‘AM’ with normal seed size (data not shown). Therefore, vegetative vigor may play a small role in the resistance to powdery mildew. However, it cannot be ruled out that these minor QTLs were detected simply coincidently at these positions.

In conclusion, a major QTL for PMR in a cultivated red fruit watermelon was detected from a genomic region carrying a cluster of NBS-LRR genes on Chr02. A major QTL controlling SS was located near the QTL for PMR on Chr02. For FS, a QTL controlling longitudinal fruit elongation was detected on Chr03, and a gene homologous to *SUN* determining FS in tomato colocalized with this QTL. The results presented herein provide useful information that is practically applicable to MAS and gene cloning for PMR and fruit-related traits.

## Supporting Information

S1 File Location of unigenes containing single nucleotide polymorphisms and insertions/deletions between ‘Arka Manik’ and ‘TS34’ on the watermelon pseudomolecule (Cucurbit Genome Database, http://www.icugi.org/cgi-bin/ICuGI/genome). (TIF)

S2 File DNA and protein sequence alignments for the NBS-LRR gene mapped at the powdery mildew-resistance (PMR) QTL. (DOCX)

S3 File DNA and protein sequence alignments for the SUN gene mapped at the fruit shape (FS) QTL. (DOCX)

S1 Table List of DNA markers used for the construction of the intraspecific genetic linkage map. (XLSX)

S2 Table List of putative disease resistance genes (R genes) retrieved from the Cucurbit Genomics Database (CuGenDB; http://www.icugi.org/cgi-bin/ICuGI/index.cgi). (XLSX)

S3 Table List of putative fruit shape genes retrieved from the Cucurbit Genomics Database (CuGenDB; http://www.icugi.org/cgi-bin/ICuGI/index.cgi). (XLSX)
