# Supplementary figures and images for: Major Quantitative Trait Loci and Putative Candidate Genes for Powdery Mildew Resistance and Fruit-Related Traits Revealed by an Intraspecific Genetic Map for Watermelon (Citrullus lanatus var. lanatus)
Source: PLoS One. 2015 Dec 23;10(12):e0145665. doi: 10.1371/journal.pone.0145665 (PMC4689417; doi:10.1371/journal.pone.0145665)

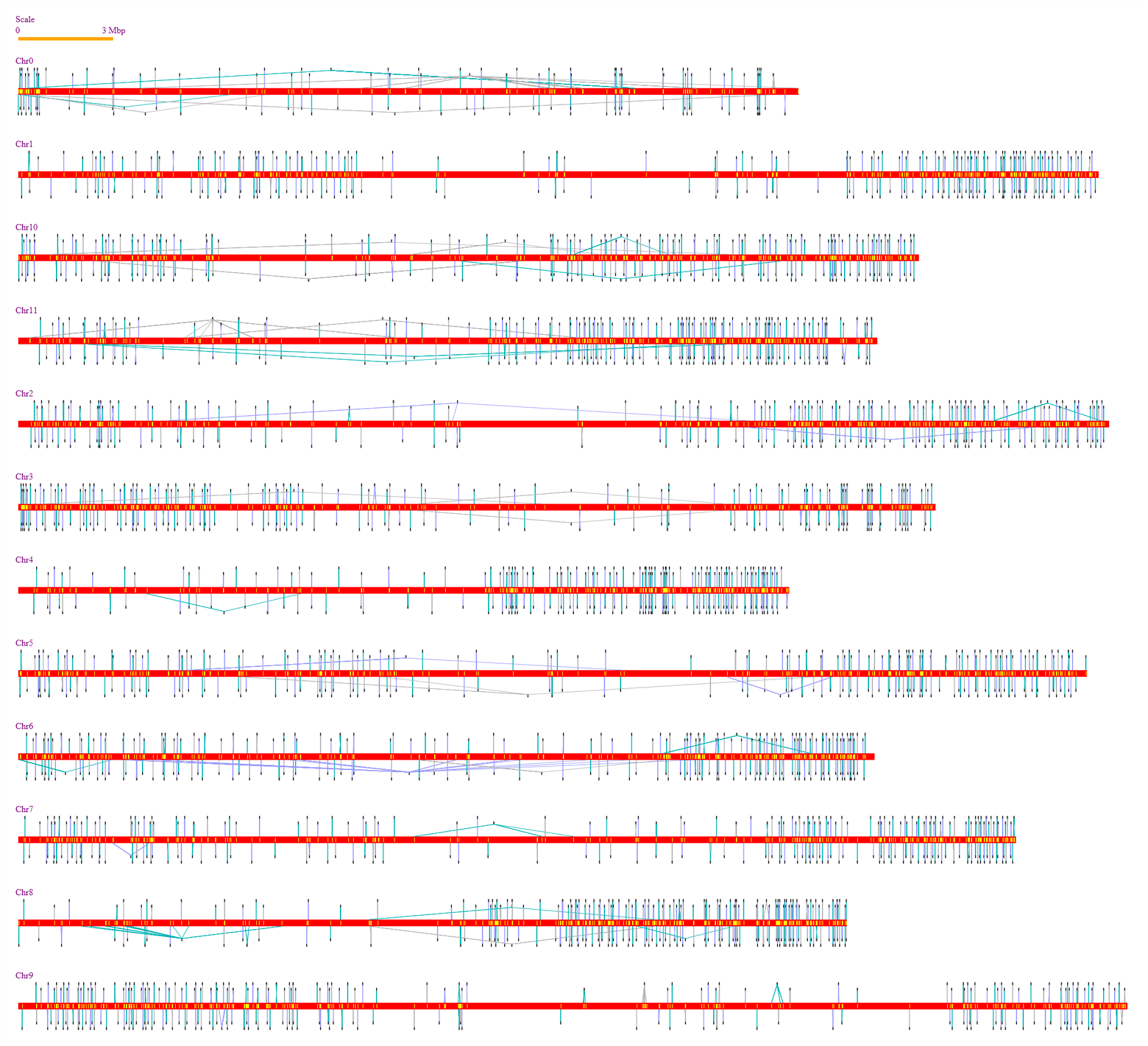

Supplement: S1 File — (TIF) [file pone.0145665.s001.tif]
