# Supplementary material for: Major Quantitative Trait Loci and Putative Candidate Genes for Powdery Mildew Resistance and Fruit-Related Traits Revealed by an Intraspecific Genetic Map for Watermelon (Citrullus lanatus var. lanatus)
Source: PLoS One. 2015 Dec 23;10(12):e0145665. doi: 10.1371/journal.pone.0145665 (PMC4689417; doi:10.1371/journal.pone.0145665)
Supplement: S2 File — (DOCX) [file pone.0145665.s002.docx]

S2 File.

DNA and protein Sequence alignment for NBS-LRR gene mapped at the powdery mildew-resistance (PMR) QTL.

**<DNA sequence_Cla019831>**

AM_chr2_cla019831 ATGGCGGGATCGTTAGTTGGTGGCGCGGCATTGGGTGTTCCATTTAACGAGTTGGCAGTC60

TS_chr2_cla019831 ATGGCGGGATCGTTAGTTGGTGGCGCGGCATTGGGTGTTCCATTTAACGAGTTGGCAGTC60

************************************************************

AM_chr2_cla019831 ATCTTGAAGAATTTTGGCGAAAGGGCGATCAGTTTTAATCCTGTTCTTAAGGAGACCCAA120

TS_chr2_cla019831 ATCTTGAAGAATTTTGGCGAAAGGGCGATCAGTTTTAATCCTGTTCTTAAGGAGACCCAA120

************************************************************

AM_chr2_cla019831 TCCAAGGTAAGTGATATAATTCCTCTGGTTAAAGAAATAGATGAGCTTAATGAAGCCCTG180

Ts_chr2_cla019831 TCCAAGGTAAGTGATATAATTCCTCTGGTTAAAGAAATAGATGAGCTTAATGAAGCCCTG180

************************************************************

AM_chr2_cla019831 GAGTATCCAAAAGAAGAAACGGAGAAGTTAAGAAACCTATTAGAAGATGCTAAAAAGCTA240

Ts_chr2_cla019831 GAGTATCCAAAAGAAGAAACGGAGAAGTTAAGAAACCTATTAGAAGATGCTAAAAAGCTA240

************************************************************

AM_chr2_cla019831 CTTCTTCGGTGTGTAAGATTGAGGAAGCTTGATTATATAAGGAAATCAACTCACACAGAG300

Ts_chr2_cla019831 CTTCTTCGGTGTGTAAGATTGAGGAAGCTTGATTATATAAGGAAATCAACTCACACAGAG300

************************************************************

AM_chr2_cla019831 AAGCTTCGTGATTTGAATACCAAAATTGCAAGTTTCAAGGAGGTTTTGTTGATGCAAACG360

Ts_chr2_cla019831 AAGCTTCGTGATTTGAATACCAAAATTGCAAGTTTCAAGGAGGTTTTGTTGATGCAAACG360

************************************************************

AM_chr2_cla019831 GCTAGAGACGGGAAGAAAACCTTGAAGATAGCGTCTGAAATCAAGGACGTCGTTCTGAGG420

Ts_chr2_cla019831 GCTAGAGACGGGAAGAAAACCTTGAAGATAGCGTCTGAAATCAAGGACGTCGTTCTGAGG420

************************************************************

AM_chr2_cla019831 CTTGATAGCAAGTCTGGATCGAGCAATCCGGTGGATTTAATTGTGAAGGTTCCTGAGATT480

Ts_chr2_cla019831 CTTGATAGCAAGTCTGGATCGAGCAATCCGGTGGATTTAATTGTGAAGGTTCCTGAGATT480

************************************************************

AM_chr2_cla019831 ACAGAAGAAAGTGTTGGATTGGAAACGCCAGTTGAGAAATTGAAGGCGAAACTTTTCAAA540

Ts_chr2_cla019831 ACAGAAGAAAGTGTTGGATTGGAAACGCCAGTTGAGAAATTGAAGGCGAAACTTTTCAAA540

************************************************************

AM_chr2_cla019831 GATGGGGTTCGATTGTTGGTATTGACAGCTCCCGGAGGTTGTGGAAAAAGCACTCTGGCC600

Ts_chr2_cla019831 GATGGGGTTCGATTGTTGGTATTGACAGCTCCCGGAGGTTGTGGAAAAAGCACTCTGGCC600

************************************************************

AM_chr2_cla019831 GTAAGATTTTGTCACGACAAGCAAGTTAAAAATAAATTTCAGAGAAACATCTTTTTCCTC660

Ts_chr2_cla019831 GTAAGATTTTGTCACGACAAGCAAGTTAAAAATAAATTTCAGAGAAACATCTTTTTCCTC660

************************************************************

AM_chr2_cla019831 ACTGTTTCAAGCAAACCTGATACGAAACTCATCTTAAAATCTATAATTCAAAGCCTCGGG720

Ts_chr2_cla019831 ACTGTTTCAAGCAAACCTGATACGAAACTCATCTTAAAATCTATAATTCAAAGCCTCGGG720

************************************************************

AM_chr2_cla019831 AGGCCTGTAGTATCAGATACTGTAAGGGACGATGAGGCATTCCGGTGTTTAGAACTACTG780

Ts_chr2_cla019831 AGGCCTGTAGTATCAGATACTGTAAGGGACGATGAGGCATTCCGGTGTTTAGAACTACTG780

************************************************************

AM_chr2_cla019831 GTGGGGCAATCGAGTCCAAATCCTGTATTGATAGTGTTGGACGATGTCTGGGAAGGTTCC840

Ts_chr2_cla019831 GTGGGGCAATCGAGTCCAAATCCTGTATTGATAGTGTTGGACGATGTCTGGGAAGGTTCC840

************************************************************

AM_chr2_cla019831 GAATCAAATAAGCTTCTTGAAAAGTTCTCCCGAATGCCTAACTGCAAAATTTTGGTCACC900

Ts_chr2_cla019831 GAATCAAATAAGCTTCTTGAAAAGTTCTCCCGAATGCCTAACTGCAAAATTTTGGTCACC900

************************************************************

AM_chr2_cla019831 TCAAGATTCAAGTTTCCTGCATTTGGTGAGTCATATGATTTGGAACCTTTGAACCATAAG960

Ts_chr2_cla019831 TCAAGATTCAAGTTTCCTGCATTTGGTGAGTCATATGATTTGGAACCTTTGAACCATAAG960

************************************************************

AM_chr2_cla019831 GATGCAAAGGAGCTGTTTCATCGCTCGGCATCGCTGGATAACAGAATGCCACAGCTCCCA1020

Ts_chr2_cla019831 GATGCAAAGGAGCTGTTTCATCGCTCGGCATCGCTGGATAACAGAATGCCACAGCTCCCA1020

************************************************************

AM_chr2_cla019831 GATGATGAAATTGTAGAAAAGATAGTGAGGGGTTGTAAGAGATTCCCACTTGCACTGAAA1080

Ts_chr2_cla019831 GATGATGAAATTGTAGAAAAGATAGTGAGGGGTTGTAAGAGATTCCCACTTGCACTGAAA1080

************************************************************

AM_chr2_cla019831 GTGATTGCAAGATCACTTTCGGG**T**AGAGCTACTTCGGTTTGGAAAGTTACAGAGAGGAAA1140

Ts_chr2_cla019831 GTGATTGCAAGATCACTTTCGGG**G**AGAGCTACTTCGGTTTGGAAAGTTACAGAGAGGAAA1140

*********************** ************************************

AM_chr2_cla019831 TTATCTAGAGGAGATTCTATTCTGGGTTCTGAGAAAGAGCTTCTGGAGTGCCTCAAAGGC1200

Ts_chr2_cla019831 TTATCTAGAGGAGATTCTATTCTGGGTTCTGAGAAAGAGCTTCTGGAGTGCCTCAAAGGC1200

************************************************************

AM_chr2_cla019831 ACCTTAGATGCAGTCCCAGATGACAAGATGGTGCTCAAGGAGTGTTTCATGGACTTGGGT1260

Ts_chr2_cla019831 ACCTTAGATGCAGTCCCAGATGACAAGATGGTGCTCAAGGAGTGTTTCATGGACTTGGGT1260

************************************************************

AM_chr2_cla019831 TCATTTCCTGAAGACCAGAGAATTCGTGTGACTACCTTCAT**T**GACATTTGTGCAGTGTTG1320

Ts_chr2_cla019831 TCATTTCCTGAAGACCAGAGAATTCGTGTGACTACCTTCAT**C**GACATTTGTGCAGTGTTG1320

***************************************** ******************

AM_chr2_cla019831 TATGAACAAGATGAATGTGAAACAATGTCAAACCTTGATGAGCTCTTCACCCGGACTTTA1380

Ts_chr2_cla019831 TATGAACAAGATGAATGTGAAACAATGTCAAACCTTGATGAGCTCTTCACCCGGACTTTA1380

************************************************************

AM_chr2_cla019831 GTTAACACTATCTCTTTGAGAAACGAAGCACATGAAGACGATTACTACAGTGAGTCCTAT1440

Ts_chr2_cla019831 GTTAACACTATCTCTTTGAGAAACGAAGCACATGAAGACGATTACTACAGTGAGTCCTAT1440

************************************************************

AM_chr2_cla019831 ATTACACAGCATGACATACTTAGAGAGTTGGCTGTCCATTTGACTAATGAGCAGCGCATA1500

Ts_chr2_cla019831 ATTACACAGCATGACATACTTAGAGAGTTGGCTGTCCATTTGACTAATGAGCAGCGCATA1500

************************************************************

AM_chr2_cla019831 GACCAAAGAACAAGATTGCTTGTGGATATTAATAGAAATGAATTTCCCAAATGGTGGTCT1560

Ts_chr2_cla019831 GACCAAAGAACAAGATTGCTTGTGGATATTAATAGAAATGAATTTCCCAAATGGTGGTCT1560

************************************************************

AM_chr2_cla019831 GAAAAAGAGATGCAACCTGTGAATGCCCGCCTTTTGTCCATAACAACAGATGAGATGTTC1620

Ts_chr2_cla019831 GAAAAAGAGATGCAACCTGTGAATGCCCGCCTTTTGTCCATAACAACAGATGAGATGTTC1620

************************************************************

AM_chr2_cla019831 TCATCATGTTGGCCTGACATGGAAGTACCTGAAGTTGAGGTGTTAATTCTAAATCCTGGG1680

Ts_chr2_cla019831 TCATCATGTTGGCCTGACATGGAAGTACCTGAAGTTGAGGTGTTAATTCTAAATCCTGGG1680

************************************************************

AM_chr2_cla019831 TCAAAAACTTACAAATTGCCCCAGTTCGTGAAGAGAATGAACAGATTGAAAGCTCTGATT1740

Ts_chr2_cla019831 TCAAAAACTTACAAATTGCCCCAGTTCGTGAAGAGAATGAACAGATTGAAAGCTCTGATT1740

************************************************************

AM_chr2_cla019831 GTCAGGAATTACAGGTCCTTTCCAACTGAATTGACAAGTGATTATCAACTAATCAACTGT1800

Ts_chr2_cla019831 GTCAGGAATTACAGGTCCTTTCCAACTGAATTGACAAGTGATTATCAACTAATCAACTGT1800

************************************************************

AM_chr2_cla019831 TTGTCAAGGCTAGAAAGAATCAGTCTTGAGCGAATTTCAATTTCTTCATTTAGTGACCAC1860

Ts_chr2_cla019831 TTGTCAAGGCTAGAAAGAATCAGTCTTGAGCGAATTTCAATTTCTTCATTTAGTGACCAC1860

************************************************************

AM_chr2_cla019831 AACCTGAAACCACTGTTACATCTTAAGAAGCTATCGTTCTTTATGTGCAAACTTGATAGA1920

Ts_chr2_cla019831 AACCTGAAACCACTGTTACATCTTAAGAAGCTATCGTTCTTTATGTGCAAACTTGATAGA1920

************************************************************

AM_chr2_cla019831 GCTTTCACGAACTGCTCAACCCAGATCTCATCCATGTTGCCTAACTTACTTGAGATTTCC1980

Ts_chr2_cla019831 GCTTTCACGAACTGCTCAACCCAGATCTCATCCATGTTGCCTAACTTACTTGAGATTTCC1980

************************************************************

AM_chr2_cla019831 ATAGATTTTTGCAACGATTTGGTGGCTCTCCCTGTTGGACTATGTGAAATTATCACATTG2040

Ts_chr2_cla019831 ATAGATTTTTGCAACGATTTGGTGGCTCTCCCTGTTGGACTATGTGAAATTATCACATTG2040

************************************************************

AM_chr2_cla019831 GAGAAGCTGAGCATTACAAACTGTCATGGACTATCTTCGTTACCAGAGGAAATTGGGCAG2100

Ts_chr2_cla019831 GAGAAGCTGAGCATTACAAACTGTCATGGACTATCTTCGTTACCAGAGGAAATTGGGCAG2100

************************************************************

AM_chr2_cla019831 TTGATTAATCTAAAAACTCTAAGGCTTAGATCTTGTATTCATTTGGAGAAGTTGCCAGAA2160

Ts_chr2_cla019831 TTGATTAATCTAAAAACTCTAAGGCTTAGATCTTGTATTCATTTGGAGAAGTTGCCAGAA2160

************************************************************

AM_chr2_cla019831 TCAATCTCAAGGCTCCGAGAATTAGTGGATCTTGACATATCTCATTGTGTTGGCCTTACC2220

Ts_chr2_cla019831 TCAATCTCAAGGCTCCGAGAATTAGTGGATCTTGACATATCTCATTGTGTTGGCCTTACC2220

************************************************************

AM_chr2_cla019831 AAACTGCCAGATAAGATTGGCAACTTGCAGAAGTTGGAAAAGCTTAATATGTGGAGTTGT2280

Ts_chr2_cla019831 AAACTGCCAGATAAGATTGGCAACTTGCAGAAGTTGGAAAAGCTTAATATGTGGAGTTGT2280

************************************************************

AM_chr2_cla019831 CCGATCATGCACAAGCTTCCAGGATCTCTAAGAAATCTAAAAAACATGAAGAAAGTAGTT2340

Ts_chr2_cla019831 CCGATCATGCACAAGCTTCCAGGATCTCTAAGAAATCTAAAAAACATGAAGAAAGTAGTT2340

************************************************************

AM_chr2_cla019831 TGTGAAGGGGAGATTGCAAAATGGGTTAATATTGTCTCACCTCGGCTTCGCAATGTGGTA2400

Ts_chr2_cla019831 TGTGAAGGGGAGATTGCAAAATGGGTTAATATTGTCTCACCTCGGCTTCGCAATGTGGTA2400

************************************************************

AM_chr2_cla019831 AAAGAACACAAGGAAGAAGTCAACTTGGATTGGCTATTAGCATGA2445

Ts_chr2_cla019831 AAAGAACACAAGGAAGAAGTCAACTTGGATTGGCTATTAGCATGA2445

*********************************************

**<DNA sequence_Cla019844>**

AM_chr2_cla019844 ATGGAGATTGTTATTTCAGTTGCTGCAAAAGCTGCTGAGTACACAGTTAAACCCATTGGA 60

TS_chr2_cla019844 ATGGAGATTGTTATTTCAGTTGCTGCAAAAGCTGCTGAGTACACAGTTAAACCCATTGGA 60

************************************************************

AM_chr2_cla019844 CGTTGGATGGGTTATCTGATTTTCTACCAAAACAATGTTAAGGATCTTGAATACCAACTT 120

TS_chr2_cla019844 CGTTGGATGGGTTATCTGATTTTCTACCAAAACAATGTTAAGGATCTTGAATACCAACTT 120

************************************************************

AM_chr2_cla019844 AGAGCCCTAGAAGATACCAGATGCAGGGTGCAGAATATGGTTGATGAAGCAAGAAGAAAT 180

TS_chr2_cla019844 AGAGCCCTAGAAGATACCAGATGCAGGGTGCAGAATATGGTTGATGAAGCAAGAAGAAAT 180

************************************************************

AM_chr2_cla019844 GCTGATGATATAGAAGTTGACGTTCAGGAATGGCTAACCAAGGTGGATAAGCTCAACAAT 240

TS_chr2_cla019844 GCTGATGATATAGAAGTTGACGTTCAGGAATGGCTAACCAAGGTGGATAAGCTCAACAAT 240

************************************************************

AM_chr2_cla019844 GAAATTGAGATTTCCCATTTAGATGAAAACCAAACAAAGAATAAATGCTGCATTGGTTTA 300

TS_chr2_cla019844 GAAATTGAGATTTCCCATTTAGATGAAAACCAAACAAAGAATAAATGCTGCATTGGTTTA 300

************************************************************

AM_chr2_cla019844 TTCTTGGTTTTTGTCCAGCGGTACCGGTTAAGCAAAAAAGCAAAGATAGAGGCATCCAAT 360

TS_chr2_cla019844 TTCTTGGTTTTTGTCCAGCGGTACCGGTTAAGCAAAAAAGCAAAGATAGAGGCATCCAAT 360

************************************************************

AM_chr2_cla019844 GTCAAAAAGATGAAGGAGGAAGGAAAATTTGACAAAGTTTCCTATCGTGGAGTTCTACCA 420

TS_chr2_cla019844 GTCAAAAAGATGAAGGAGGAAGGAAAATTTGACAAAGTTTCCTATCGTGGAGTTCTACCA 420

************************************************************

AM_chr2_cla019844 GGGGTGGGAAATTCAGCTATCAAAGGTTTCCTAACCTTTGGATCAAGAACATCCGTTGTG 480

TS_chr2_cla019844 GGGGTGGGAAATTCAGCTATCAAAGGTTTCCTAACCTTTGGATCAAGAACATCCGTTGTG 480

************************************************************

AM_chr2_cla019844 AAGGAGGTTATTGAGGCACTGATGGATTCTAAAGTCAGCATGGTTGGATTGTATGGGATG 540

TS_chr2_cla019844 AAGGAGGTTATTGAGGCACTGATGGATTCTAAAGTCAGCATGGTTGGATTGTATGGGATG 540

************************************************************

AM_chr2_cla019844 GGTGGTGTTGGCAAAACTATGTTAGTCAAAGAAATTTCAAGACAAGTTAGGGAGGTCAGA 600

TS_chr2_cla019844 GGTGGTGTTGGCAAAACTATGTTAGTCAAAGAAATTTCAAGACAAGTTAGGGAGGTCAGA 600

************************************************************

AM_chr2_cla019844 TTACTTGATGAAGTGGTTATGGTAACTATAGGCCAGACTCCAGATATTAGAAGTATTCAG 660

TS_chr2_cla019844 TTACTTGATGAAGTGGTTATGGTAACTATAGGCCAGACTCCAGATATTAGAAGTATTCAG 660

************************************************************

AM_chr2_cla019844 GCAAAAATCGGTGACATGCTAAGCTTGTCTTTTCAACAAGAAAGTGTTGAAGGAAGAGCA 720

TS_chr2_cla019844 GCAAAAATCGGTGACATGCTAAGCTTGTCTTTTCAACAAGAAAGTGTTGAAGGAAGAGCA 720

************************************************************

AM_chr2_cla019844 GCCCTATTACAAAAGAGGTTGAAGAAGGAGAAAAAGATCCTTATAGTGTTAGATGATATG 780

TS_chr2_cla019844 GCCCTATTACAAAAGAGGTTGAAGAAGGAGAAAAAGATCCTTATAGTGTTAGATGATATG 780

************************************************************

AM_chr2_cla019844 TGGGAGGGACTTGATTTAGAAACTATTGGAATTCCTTACGGGGAAGATCATGAAGGATGT 840

TS_chr2_cla019844 TGGGAGGGACTTGATTTAGAAACTATTGGAATTCCTTACGGGGAAGATCATGAAGGATGT 840

************************************************************

AM_chr2_cla019844 AAGATACTTATCACTTCAAGACATCACAATGTATTATACAATAAAATGTATACTCGTAAT 900

TS_chr2_cla019844 AAGATACTTATCACTTCAAGACATCACAATGTATTATACAATAAAATGTATACTCGTAAT 900

************************************************************

AM_chr2_cla019844 AATTTTGAGGTAAAGTTTCTTAGTGAAGAAGAATCATGGAGTTTCTTCAAAAGCATGGTA 960

TS_chr2_cla019844 AATTTTGAGGTAAAGTTTCTTAGTGAAGAAGAATCATGGAGTTTCTTCAAAAGCATGGTA 960

************************************************************

AM_chr2_cla019844 GGTGAATCACTTGAAATTCCAGTCTTGAAATCTGTAGCATCCAAGGTAGCGAAGGAATGT 1020

TS_chr2_cla019844 GGTGAATCACTTGAAATTCCAGTCTTGAAATCTGTAGCATCCAAGGTAGCGAAGGAATGT 1020

************************************************************

AM_chr2_cla019844 GCAGGGTTGCCAATTGCACTCAGTACAGTTGCGAAGGCATTGAGTGGAAAATCTTTGCCG 1080

TS_chr2_cla019844 GCAGGGTTGCCAATTGCACTCAGTACAGTTGCGAAGGCATTGAGTGGAAAATCTTTGCCG 1080

************************************************************

AM_chr2_cla019844 ATATGGAGGGATGCCTTAAAACAACTGCAAAATCCTGCTGCAGTAAATGAAGGGGTGGGC 1140

TS_chr2_cla019844 ATATGGAGGGATGCCTTAAAACAACTGCAAAATCCTGCTGCAGTAAATGAAGGGGTGGGC 1140

************************************************************

AM_chr2_cla019844 AAAGAAGCTTATGCCTCAGTTGAACTAAGCTATAAGTATGTAGAAAGTGAAGAAGCAAAG 1200

TS_chr2_cla019844 AAAGAAGCTTATGCCTCAGTTGAACTAAGCTATAAGTATGTAGAAAGTGAAGAAGCAAAG 1200

************************************************************

AM_chr2_cla019844 TTGCTATTCCTACTTTGTAGTATGTTTCTTGAAGATTATGACATTAACATGGAAAAATTG 1260

TS_chr2_cla019844 TTGCTATTCCTACTTTGTAGTATGTTTCTTGAAGATTATGACATTAACATGGAAAAATTG 1260

************************************************************

AM_chr2_cla019844 CTCATTTATGCTATTGGTCTGAGATTGATACAGGGTCTCCATTCTTTGGCTGATGCAAGA 1320

TS_chr2_cla019844 CTCATTTATGCTATTGGTCTGAGATTGATACAGGGTCTCCATTCTTTGGCTGATGCAAGA 1320

************************************************************

AM_chr2_cla019844 GACAGAATGGTTAAATTGGTTGATGATCTCAAATCTAGTAGTTTACTTCTAGATTCAGAT 1380

TS_chr2_cla019844 GACAGAATGGTTAAATTGGTTGATGATCTCAAATCTAGTAGTTTACTTCTAGATTCAGAT 1380

************************************************************

AM_chr2_cla019844 CGAGGGGAAAATTTTGTTAAGATGCATGATGTTGTTCGCAATGTTGCTATTTCAATTGCA 1440

TS_chr2_cla019844 CGAGGGGAAAATTTTGTTAAGATGCATGATGTTGTTCGCAATGTTGCTATTTCAATTGCA 1440

************************************************************

AM_chr2_cla019844 TCAAGAGATGATAAGATGTGTACAATGAGTTATGGGCGAGGATCTACTGAATGGATAGAA 1500

TS_chr2_cla019844 TCAAGAGATGATAAGATGTGTACAATGAGTTATGGGCGAGGATCTACTGAATGGATAGAA 1500

************************************************************

AM_chr2_cla019844 GACGAGGCATTCAGAAAATACAATGCAGTCTTAATAAATACTGAAAACTTTCACAAGCTT 1560

TS_chr2_cla019844 GACGAGGCATTCAGAAAATACAATGCAGTCTTAATAAATACTGAAAACTTTCACAAGCTT 1560

************************************************************

AM_chr2_cla019844 CCTCAGAAGTTGATGTTTCCAAACCTTGAGCTGCTGGTACTAGTTCGAGGTACTTTTTTG 1620

TS_chr2_cla019844 CCTCAGAAGTTGATGTTTCCAAACCTTGAGCTGCTGGTACTAGTTCGAGGTACTTTTTTG 1620

************************************************************

AM_chr2_cla019844 GAACCAAATATTCAGATGCCAGAAGTTTTCTTAATGGAATTGGTAAAACTCAAAGTTTTG 1680

TS_chr2_cla019844 GAACCAAATATTCAGATGCCAGAAGTTTTCTTAATGGAATTGGTAAAACTCAAAGTTTTG 1680

************************************************************

AM_chr2_cla019844 GAGTTGCATAATTTGCAGATTTCGCTGTCATCATTCCACTCCTTGGCGAACCTTCAAACT 1740

TS_chr2_cla019844 GAGTTGCATAATTTGCAGATTTCGCTGTCATCATTCCACTCCTTGGCGAACCTTCAAACT 1740

************************************************************

AM_chr2_cla019844 TTATGTCTATGGTTTTGTGAGTTGGTGAACATGGACATGATCAAGGAGCTAAAGAAACTT 1800

TS_chr2_cla019844 TTATGTCTATGGTTTTGTGAGTTGGTGAACATGGACATGATCAAGGAGCTAAAGAAACTT 1800

************************************************************

AM_chr2_cla019844 GAAATTCTCAGCTTTAGAGGATGTAACATCAAAGAAGTACCTCCAGCTATCGGCCAATTG 1860

TS_chr2_cla019844 GAAATTCTCAGCTTTAGAGGATGTAACATCAAAGAAGTACCTCCAGCTATCGGCCAATTG 1860

************************************************************

AM_chr2_cla019844 ACACAACTCAAGTCATTAGATTTAAAATATTGCTATGAACTGGAGGTGATTCCACCTAAT 1920

TS_chr2_cla019844 ACACAACTCAAGTCATTAGATTTAAAATATTGCTATGAACTGGAGGTGATTCCACCTAAT 1920

************************************************************

AM_chr2_cla019844 GTGATCTCAAAATTGATAAAATTAGAAGAGTTGGATATGGAAGAGAGTTTTGTTGGATGG 1980

TS_chr2_cla019844 GTGATCTCAAAATTGATAAAATTAGAAGAGTTGGATATGGAAGAGAGTTTTGTTGGATGG 1980

************************************************************

AM_chr2_cla019844 GATAGGATAGGATTGACCAGTCAAAAACAGAATGCCAGCCTTTTGGAATTACAGTTT**G**TG 2040

TS_chr2_cla019844 GATAGGATAGGATTGACCAGTCAAAAACAGAATGCCAGCCTTTTGGAATTACAGTTT**T**TG 2040

********************************************************* **

AM_chr2_cla019844 ACTTCCCTTACTACTTTATATTTATGTGTTCCAGATGGTAGCGTTATACCAAAACAACTG 2100

TS_chr2_cla019844 ACTTCCCTTACTACTTTATATTTATGTGTTCCAGATGGTAGCGTTATACCAAAACAACTG 2100

************************************************************

AM_chr2_cla019844 TTTTTAGGAAATCTGAAATTAGAAAGATTTCAAATAGTCCTTGGTGCAGAATGGCCTGAA 2160

TS_chr2_cla019844 TTTTTAGGAAATCTGAAATTAGAAAGATTTCAAATAGTCCTTGGTGCAGAATGGCCTGAA 2160

************************************************************

AM_chr2_cla019844 TATACATTTAACACCTCCAAAGTGATGTATCTGAAGGTCGATTCAAAAATTATTTTTAGT 2220

TS_chr2_cla019844 TATACATTTAACACCTCCAAAGTGATGTATCTGAAGGTCGATTCAAAAATTATTTTTAGT 2220

************************************************************

AM_chr2_cla019844 GAGGGGATGAAAAGGTTACTCTGTAGATCTGAAGAATTGTACTTAGAAGTTGTCCATGGA 2280

TS_chr2_cla019844 GAGGGGATGAAAAGGTTACTCTGTAGATCTGAAGAATTGTACTTAGAAGTTGTCCATGGA 2280

************************************************************

AM_chr2_cla019844 AAGGATGTTCTTATTGAATTGGATGAAAATGATGTACCACCTTTGAGGCATCTTCACCTG 2340

TS_chr2_cla019844 AAGGATGTTCTTATTGAATTGGATGAAAATGATGTACCACCTTTGAGGCATCTTCACCTG 2340

************************************************************

AM_chr2_cla019844 TTTGATATGTTGGATACACAATATTTAATTGGTGAAAAAGCTTCACTTAAAGGTCTCACT 2400

TS_chr2_cla019844 TTTGATATGTTGGATACACAATATTTAATTGGTGAAAAAGCTTCACTTAAAGGTCTCACT 2400

************************************************************

AM_chr2_cla019844 AATTTGGAGATGTTATCTCTTAATAGAATGATGAGCTTGGAGAATACAATTCATGGGTGT 2460

TS_chr2_cla019844 AATTTGGAGATGTTATCTCTTAATAGAATGATGAGCTTGGAGAATACAATTCATGGGTGT 2460

************************************************************

AM_chr2_cla019844 ATCAAAGTTGTTCCTTTTAATAAATTGAAAATCATAAAAGTGGTGGAGTGTAAAGCATTG 2520

TS_chr2_cla019844 ATCAAAGTTGTTCCTTTTAATAAATTGAAAATCATAAAAGTGGTGGAGTGTAAAGCATTG 2520

************************************************************

AM_chr2_cla019844 AGGAATCTCTTTTTGTCATCTATCATGAGTGGCCTTTCAAGTCTTCAAACGATAGATGTT 2580

TS_chr2_cla019844 AGGAATCTCTTTTTGTCATCTATCATGAGTGGCCTTTCAAGTCTTCAAACGATAGATGTT 2580

************************************************************

AM_chr2_cla019844 TCTGGCTGTGAAATGATCGAGGCGATTGTTGGTGTGGAAGATGAGGCGACAAGCCAGTTT 2640

TS_chr2_cla019844 TCTGGCTGTGAAATGATCGAGGCGATTGTTGGTGTGGAAGATGAGGCGACAAGCCAGTTT 2640

************************************************************

AM_chr2_cla019844 GAGTGTAGTAAATTAACCTCCTTATCCTTAATTGGCTTACCTTGGCTTACAAGTTTCTGC 2700

TS_chr2_cla019844 GAGTGTAGTAAATTAACCTCCTTATCCTTAATTGGCTTACCTTGGCTTACAAGTTTCTGC 2700

************************************************************

AM_chr2_cla019844 TTGAAAGTGGAGCAGCGCAGCCAAATAATTCAGTATGATGTACAACATTGGATCCCATTT 2760

TS_chr2_cla019844 TTGAAAGTGGAGCAGCGCAGCCAAATAATTCAGTATGATGTACAACATTGGATCCCATTT 2760

************************************************************

AM_chr2_cla019844 TTCAATGAGCAGGTTGCATTTCCGAGTTTGGAAACGTTAGTACTTAGTGGCTTGCACAAA 2820

TS_chr2_cla019844 TTCAATGAGCAGGTTGCATTTCCGAGTTTGGAAACGTTAGTACTTAGTGGCTTGCACAAA 2820

************************************************************

AM_chr2_cla019844 TTGAAGACCATATGGCATAATGGTCTGACAGTGGAGTCGTTCCGCAAACTCAAAAGGATA 2880

TS_chr2_cla019844 TTGAAGACCATATGGCATAATGGTCTGACAGTGGAGTCGTTCCGCAAACTCAAAAGGATA 2880

************************************************************

AM_chr2_cla019844 CAACTTACTCATTGTAAAAGTCTTAGAATTGTGTTCCCTTCAAATACAATAAGGATGCTT 2940

TS_chr2_cla019844 CAACTTACTCATTGTAAAAGTCTTAGAATTGTGTTCCCTTCAAATACAATAAGGATGCTT 2940

************************************************************

AM_chr2_cla019844 AAGAGTCTCGAGAAGTTGGAGATACAGAACTGCGAATTGATTGAAGAGATATTTGAAATT 3000

TS_chr2_cla019844 AAGAGTCTCGAGAAGTTGGAGATACAGAACTGCGAATTGATTGAAGAGATATTTGAAATT 3000

************************************************************

AM_chr2_cla019844 CATTGGTCAAATACCAAGGAAGAAGGAGATATATTTGCCACCCAATTGAGATACCTGGAT 3060

TS_chr2_cla019844 CATTGGTCAAATACCAAGGAAGAAGGAGATATATTTGCCACCCAATTGAGATACCTGGAT 3060

************************************************************

AM_chr2_cla019844 TTAAAAGATCTACCAAGATTGAAGAATGTTTGGAACAGAGATCCACAAGAATTTGTTACC 3120

TS_chr2_cla019844 TTAAAAGATCTACCAAGATTGAAGAATGTTTGGAACAGAGATCCACAAGAATTTGTTACC 3120

************************************************************

AM_chr2_cla019844 TTTCAAAACATAGCTGGTGTCACTGTTGACGGTTGCCCTGAACTGAAAAGTCTTTTTCCA 3180

TS_chr2_cla019844 TTTCAAAACATAGCTGGTGTCACTGTTGACGGTTGCCCTGAACTGAAAAGTCTTTTTCCA 3180

************************************************************

AM_chr2_cla019844 GCCTCCTTTGCTACCAATCTTCAATTACTAGAAAGCTTAGTCTTCGAGCGTTGTGGATTG 3240

TS_chr2_cla019844 GCCTCCTTTGCTACCAATCTTCAATTACTAGAAAGCTTAGTCTTCGAGCGTTGTGGATTG 3240

************************************************************

AM_chr2_cla019844 GAACAAATCTTTGTGAAGGAAGAAAGATTTGAGACAACCAAAATACAGTTTGTCTTCCCT 3300

TS_chr2_cla019844 GAACAAATCTTTGTGAAGGAAGAAAGATTTGAGACAACCAAAATACAGTTTGTCTTCCCT 3300

************************************************************

AM_chr2_cla019844 AAGGTAATGCTTCTACATCTTACTGTTTCATTTCTGGCTTTAGACTGCACATTGGTAATG 3360

TS_chr2_cla019844 AAGGTAATGCTTCTACATCTTACTGTTTCATTTCTGGCTTTAGACTGCACATTGGTAATG 3360

************************************************************

AM_chr2_cla019844 AGAGACTTATGGTATGGTCAATCTCCCAGAGATTTGTTTCCCAGGCTTAAAGCTCTTGCA 3420

TS_chr2_cla019844 AGAGACTTATGGTATGGTCAATCTCCCAGAGATTTGTTTCCCAGGCTTAAAGCTCTTGCA 3420

************************************************************

AM_chr2_cla019844 TTGTTGGGTTCAGATAATTATAAATGGTCAATCTCAATGACTAATTTACCATTTGGACTA 3480

TS_chr2_cla019844 TTGTTGGGTTCAGATAATTATAAATGGTCAATCTCAATGACTAATTTACCATTTGGACTA 3480

************************************************************

AM_chr2_cla019844 GCTGAATTAATGTTCATTGTTGATGAACTCCATGTGATAGATTCCTTCCTGGTTGAACTC 3540

TS_chr2_cla019844 GCTGAATTAATGTTCATTGTTGATGAACTCCATGTGATAGATTCCTTCCTGGTTGAACTC 3540

************************************************************

AM_chr2_cla019844 TTTCCAAATGAAGGATTTTTCACTGCTGAGGAAAATCAAGTTGGAAGATCTGCACCTTTG 3600

TS_chr2_cla019844 TTTCCAAATGAAGGATTTTTCACTGCTGAGGAAAATCAAGTTGGAAGATCTGCACCTTTG 3600

************************************************************

AM_chr2_cla019844 AGGAGATTAAGTCTCCTTAGACTTCCTAAACTTGTACATTTATGGAAAACAAACTTCCAA 3660

TS_chr2_cla019844 AGGAGATTAAGTCTCCTTAGACTTCCTAAACTTGTACATTTATGGAAAACAAACTTCCAA 3660

************************************************************

AM_chr2_cla019844 GTAACATTTCACAATGTAGAAGTCCTCGAGGTAGAGGAATGCAGTCGATTAAATTCATTA 3720

TS_chr2_cla019844 GTAACATTTCACAATGTAGAAGTCCTCGAGGTAGAGGAATGCAGTCGATTAAATTCATTA 3720

************************************************************

AM_chr2_cla019844 TTTCCATTCTCGTTTTCTCTCCACAACTTGAAGTGTATTAGAATAGTTGCTTGTCATGGA 3780

TS_chr2_cla019844 TTTCCATTCTCGTTTTCTCTCCACAACTTGAAGTGTATTAGAATAGTTGCTTGTCATGGA 3780

************************************************************

AM_chr2_cla019844 TTGGTTCATTTGATGGATTCATCAGTAGCCAACACCCTGGTGCAGCTGCAAGAGATACAT 3840

TS_chr2_cla019844 TTGGTTCATTTGATGGATTCATCAGTAGCCAACACCCTGGTGCAGCTGCAAGAGATACAT 3840

************************************************************

AM_chr2_cla019844 TTATCTGAATGCAAAAAGATGAGCACTATAATTCCAAAAGGTGAAGCAGAAGAAGAGGGT 3900

TS_chr2_cla019844 TTATCTGAATGCAAAAAGATGAGCACTATAATTCCAAAAGGTGAAGCAGAAGAAGAGGGT 3900

************************************************************

AM_chr2_cla019844 GAAATTGTTTTCAGGCAATTGAAAATTTTGGAACTCTTTAACTTACCCAATTTATCAACC 3960

TS_chr2_cla019844 GAAATTGTTTTCAGGCAATTGAAAATTTTGGAACTCTTTAACTTACCCAATTTATCAACC 3960

************************************************************

AM_chr2_cla019844 TTTCATTCTGGGAAAAGCAATTTTAAGTTCCCATGTTTGGAAAAAGTGGTTATGAAGAAA 4020

TS_chr2_cla019844 TTTCATTCTGGGAAAAGCAATTTTAAGTTCCCATGTTTGGAAAAAGTGGTTATGAAGAAA 4020

************************************************************

AM_chr2_cla019844 TGTCCTGAAATGAAAGCATTTTCTTGTGGAGTTGTAAGTACACCTGAACATTACTGGTAT 4080

TS_chr2_cla019844 TGTCCTGAAATGAAAGCATTTTCTTGTGGAGTTGTAAGTACACCTGAACATTACTGGTAT 4080

************************************************************

AM_chr2_cla019844 GTGAAATGTGGATCAGATGAGGGGTTTTGGACAAGCAATGTTAATGCCACAATCAATCAG 4140

TS_chr2_cla019844 GTGAAATGTGGATCAGATGAGGGGTTTTGGACAAGCAATGTTAATGCCACAATCAATCAG 4140

************************************************************

AM_chr2_cla019844 CTTTGGGAGGATAATCACCTCGATTCCAGCCTGCAGAGTTTATTTACTGAAGAGGTATGT 4200

TS_chr2_cla019844 CTTTGGGAGGATAATCACCTCGATTCCAGCCTGCAGAGTTTATTTACTGAAGAGGTATGT 4200

************************************************************

AM_chr2_cla019844 ATGTCCGTTGAATCTACAAGTTTTTAG-- 4227

TS_chr2_cla019844 ATGTCCGTTGAATCTACAAGTTTTTAG-- 4227

***************************

**<Protein sequence_Cla019831>** RPW8, AAA (XP_008455702.1: melon)

AM_cla019831 MAGSLVGGAALGVPFNELAVILKNFGERAISFNPVLKETQSKVSDIIPLVKEIDELNEAL 60

Ts_cla019831 MAGSLVGGAALGVPFNELAVILKNFGERAISFNPVLKETQSKVSDIIPLVKEIDELNEAL 60

XP_008455702.1 MAGALIGGAALGVPFNELATILKNFGERAMSFDSVLKETESKVNDIIPLVKEIDDLNEFL 60

***:*:*************.*********:**:.*****:***.**********:*** *

AM_cla019831 EYPKEETEKLRNLLEDAKKLLLRCVRLRKLDYIRKSTHTEKLRDLNTKIASFKEVLLMQT 120

Ts_cla019831 EYPKEETEKLRNLLEDAKKLLLRCVRLRKLDYIRKSTHTEKLRDLNTKIASFKEVLLMQT 120

XP_008455702.1 EYPKEETEKLKNLLEDAKKLLSRCLRVGKVDLLRKSSHTEKLRELNTKIGSFKDVVLLQT 120

**********:********** **:*: *:* :***:******:*****.***:*:*:**

AM_cla019831 ARDGKKTLKIASEIKDVVLRLDSKSGSSNPVDLIVKVPEITEESVGLETPVEKLKAKLFK 180

Ts_cla019831 ARDGKKTLKIASEIKDVVLRLDSKSGSSNPVDLIVKVPEITEESVGLETPVEKLKAKLFK 180

XP_008455702.1 SRDGKKTLRLASEIKDVVLRLDGKSGLSNPVDLVVTVPVITEESVGLEKPVEKLKAKLFK 180

:*******::************.*** ******:*.** *********.***********

AM_cla019831 DGVRLLVLTAPGGCGKSTLAVRFCHDKQVKNKFQRNIFFLTVSSKPDTKLILKSIIQSLG 240

Ts_cla019831 DGVRLLVLTAPGGCGKSTLAVRFCHDKQVKNKFQRNIFFLTVSSKPDTKLILKSIIQSLG 240

XP_008455702.1 DGVRLLVVTAPGGCGKSTLAEKFCHDKQVKNKFQRNILFLVVSSKPETKRILKSIIQRLG 240

*******:************ :***************:**.*****:** ******* **

AM_cla019831 RPVVSDTVRDDEAFRCLELLVGQSSPNPVLIVLDDVWEGSESNKLLEKFSRMPNCKILVT 300

Ts_cla019831 RPVVSDTVRDDEAFRCLELLVGQSSPNPVLIVLDDVWEGSESNKLLEKFSRMPNCKILVT 300

XP_008455702.1 GPVVSDSVRDDEAFRLLELLVGQLSPNPVLIVLDDVWDGSESNKLLEKFSRLPNCKILVT 300

*****:******** ******* *************:*************:********

AM_cla019831 SRFKFPAFGESYDLEPLNHKDAKELFHRSASLDNRMPQLPDDEIVEKIVRGCKRFPLALK 360

Ts_cla019831 SRFKFPAFGESYDLEPLNHKDAKELFHRSASLDNRMPQLPDDEIVEKIVRGCKRFPLALK 360

XP_008455702.1 SRFKFPAFGESYDLEPLDHKDAKELFHRWASRGNRMLQFPDEKIVEKIVRGCKRFPLALK 360

*****************:********** ** .*** *:**::*****************

AM_cla019831 VIARSLSGRATSVWKVTERKLSRGDSILGSEKELLECLKGTLDAVPDDKMVLKECFMDLG 420

Ts_cla019831 VIARSLSGRATSVWKVTERKLSRGDSILGSEKELLECLKGTLDAVPDDKMVLKECFMDLG 420

XP_008455702.1 VIAGSLSGRATSVWEVTERKLSRGDSILGSENELLKCLKDTLDAVPDDKIVLKECFMDLG 420

*** **********:****************:***:***.*********:**********

AM_cla019831 SFPEDQRIRVTTFIDICAVLYEQDECETMSNLDELFTRTLVNTISLRNEAHEDDYYSESY 480

Ts_cla019831 SFPEDQRIRVTTFIDICAVLYEQDECETMSNLDELFTRTLVNTISLRNEAHEDDYYSESY 480

XP_008455702.1 SFPEDQRIRAATFIDMCAVLYEQDECETMSNLDELFTRTLVNTVSLRNEAHEDDYYSESY 480

*********.:****:***************************:****************

AM_cla019831 ITQHDILRELAVHLTNEQRIDQRTRLLVDINRNEFPKWWSEKEMQPVNARLLSITTDEMF 540

Ts_cla019831 ITQHDILRELAVHLTNEQRIDQRTRLLVDINRNEFPKWWSEKEMQPVNARLLSITTDEMF 540

XP_008455702.1 ITQHDILRELAVHLTNEQQVDQRTRLLVDINKNEFPKWWSEKETQPVKARLLSITTDEKF 540

******************::***********:*********** ***:********** *

AM_cla019831 SSCWPDMEVPEVEVLILNPGSKTYKLPQFVKRMNRLKALIVRNYRSFPTELTSDYQLINC 600

Ts_cla019831 SSCWPDMEVPEVEVLILNPGSKTYKLPQFVKRMNRLKALIVRNYRSFPTELTSDYQLINC 600

XP_008455702.1 SSYWPDMEAPEVEVLILNPGSETYKLPEFAKKMNRLKVLIVRNYRSFPTELTSDYQLINC 600

** *****.************:*****:*.*:*****.**********************

AM_cla019831 LSRLERISLERISISSFSDHNLKPLLHLKKLSFFMCKLDRAFTNCSTQISSMLPNLLEIS 660

Ts_cla019831 LSRLERISLERISISSFSDHNLKPLLHLKKLSFFMCKLDRAFTNCSTQISSMLPNLLEIS 660

XP_008455702.1 LSRLERISLERISISSFSDHNLKPLLHLKKLSFFMCKIDKAFTNCSTQISSILPNLLEIS 660

*************************************:*:***********:********

AM_cla019831 IDFCNDLVALPVGLCEIITLEKLSITNCHGLSSLPEEIGQLINLKTLRLRSCIHLEKLPE 720

Ts_cla019831 IDFCNDLVALPVGLCEIITLEKLSITNCHGLSSLPEEIGQLINLKTLRLRSCIHLEKLPE 720

XP_008455702.1 IDFCNDLVALPVGLCEIFTLEKLSITNCHGLSSLPEEIGQLINLKILRLRSCIHLEKLPE 720

*****************:*************************** **************

AM_cla019831 SISRLRELVDLDISHCVGLTKLPDKIGNLQKLEKLNMWSCPIMHKLPGSLRNLKNMKKVV 780

Ts_cla019831 SISRLRELVDLDISHCVGLTKLPDKIGNLQKLEKLNMWSCPIMHKLPGSLRNLKNMKKVV 780

XP_008455702.1 SISRLQELVDLDISHCVGLTKLPDKIGNLQKLEKLNMWSCPNMHKLPKSVRNLKSLKKVV 780

*****:*********************************** ***** *:****.:****

AM_cla019831 CEGEIAKWVNIVSPRLRNVVKEHKEEVNLDWLLA 814

Ts_cla019831 CEGEIAKWVNIVSPRLRNVVKEHKEEVNLDWLLA 814

XP_008455702.1 CEREIAKWVNFVSPRLGNVVKEQKEEVNLDWLYA 814

** *******:***** *****:********* *
