# Supplementary material for: Major Quantitative Trait Loci and Putative Candidate Genes for Powdery Mildew Resistance and Fruit-Related Traits Revealed by an Intraspecific Genetic Map for Watermelon (Citrullus lanatus var. lanatus)
Source: PLoS One. 2015 Dec 23;10(12):e0145665. doi: 10.1371/journal.pone.0145665 (PMC4689417; doi:10.1371/journal.pone.0145665)
Supplement: S3 File — (DOCX) [file pone.0145665.s003.docx]

S3 File.

DNA and protein Sequence alignment for the SUN gene mapped at fruit shape (FS) QTL.

**<SUN protein sequence_Cla011257>**

AM_cla011257 ATGGCCATGGATCCCAACCCCAAAAGTTTCCCGATCCTTTCTTACGTCATGGCCAGAATC 60

TS_cla011257 ATGGCCATGGATCCCAACCCCAAAAGTTTCCCGATCCTTTCTTACGTCATGGCCAGAATC 60

************************************************************

AM_cla011257 CCCTCTCTCAGCCCTCGAACGCCGGCCACTGAATTCGACATCGAACAACCTGCATCTCCG 120

TS_cla011257 CCCTCTCTCAGCCCTCGAACGCCGGCCACTGAATTCGACATCGAACAACCTGCATCTCCG 120

************************************************************

AM_cla011257 GCCTCCCGCCGC**A**CTCCTTCCGATCCTTCCTCATCCTCCTCCCGAATCGTCCATGATATG 180

TS_cla011257 GCCTCCCGCCGC**G**CTCCTTCCGATCCTTCCTCATCCTCCTCCCGAATCGTCCATGATATG 180

************ ***********************************************

AM_cla011257 CCTCATCTCTCCGACCCCAAGGTTTTGGCTTCTATGACTACCGCTATATCTGATGTTGCT 240

TS_cla011257 CCTCATCTCTCCGACCCCAAGGTTTTGGCTTCTATGACTACCGCTATATCTGATGTTGCT 240

************************************************************

AM_cla011257 CAGACCCGATCCGTCCTCAAAACCCTTGGGGAGAGACCAGACCATGAGGCTGTTGATACT 300

TS_cla011257 CAGACCCGATCCGTCCTCAAAACCCTTGGGGAGAGACCAGACCATGAGGCTGTTGATACT 300

************************************************************

AM_cla011257 GCCAAGGCTAAGCTCGCTGAGGTAGAAGTTAATTTGTCTGCCAAGCTTCAGGAAATCGTG 360

TS_cla011257 GCCAAGGCTAAGCTCGCTGAGGTAGAAGTTAATTTGTCTGCCAAGCTTCAGGAAATCGTG 360

************************************************************

AM_cla011257 CTTTCGTCTAGGCCGGCGGATGTCGAGTTGCTTGAGTGGAGGGCGCATCTTGCTGAGAAA 420

TS_cla011257 CTTTCGTCTAGGCCGGCGGATGTCGAGTTGCTTGAGTGGAGGGCGCATCTTGCTGAGAAA 420

************************************************************

AM_cla011257 GAGAACGAGTGCCGCCAGGCGGCGGATAAAGAGAAGCAGGGGTATAAGGCAATTGTGCAA 480

TS_cla011257 GAGAACGAGTGCCGCCAGGCGGCGGATAAAGAGAAGCAGGGGTATAAGGCAATTGTGCAA 480

************************************************************

AM_cla011257 CTGGATGAGATGCACGAGGCGTATGAGAAGATGTTGAAGGAAGCAGAGGAGAGGTTGGTG 540

TS_cla011257 CTGGATGAGATGCACGAGGCGTATGAGAAGATGTTGAAGGAAGCAGAGGAGAGGTTGGTG 540

************************************************************

AM_cla011257 AAGATTTACGAGTCGGCTGAGAGAGGGCTGCAGGAGGAAGAACACTTGGATCCAGTTAGC 600

TS_cla011257 AAGATTTACGAGTCGGCTGAGAGAGGGCTGCAGGAGGAAGAACACTTGGATCCAGTTAGC 600

************************************************************

AM_cla011257 GAGGAGGTCAATGAGGAGGTTGCGAGGATTCTTCAGGACGCGAATGAGAAGGAAATGGAT 660

TS_cla011257 GAGGAGGTCAATGAGGAGGTTGCGAGGATTCTTCAGGACGCGAATGAGAAGGAAATGGAT 660

************************************************************

AM_cla011257 CGAATTAGTCTCTCCGGCCGACGCTTGCGGTTCTTGCCTGAAGGATTTGGACGCATTCGG 720

TS_cla011257 CGAATTAGTCTCTCCGGCCGACGCTTGCGGTTCTTGCCTGAAGGATTTGGACGCATTCGG 720

************************************************************

AM_cla011257 GGATTGGTTGTGCTTGATATCTCCAGCAATCAACTACAGATTATCCCCGATTCAATATCT 780

TS_cla011257 GGATTGGTTGTGCTTGATATCTCCAGCAATCAACTACAGATTATCCCCGATTCAATATCT 780

************************************************************

AM_cla011257 GGCTTAGAAAATCTTGAGGAGCTGAATGCCTCATCCAATCTCCTGGAGTCACTGCCCGAC 840

TS_cla011257 GGCTTAGAAAATCTTGAGGAGCTGAATGCCTCATCCAATCTCCTGGAGTCACTGCCCGAC 840

************************************************************

AM_cla011257 TCAATTGGGTTATTACAAAAGCTGAAACTCCTGAATGTCTCTGGGAACAAGTTGCATGCC 900

TS_cla011257 TCAATTGGGTTATTACAAAAGCTGAAACTCCTGAATGTCTCTGGGAACAAGTTGCATGCC 900

************************************************************

AM_cla011257 CTTCCTGACACAATATGCCATTGCAGGTCTTTGGTGGAGTTGGATGTGAGCTTCAACAGT 960

TS_cla011257 CTTCCTGACACAATATGCCATTGCAGGTCTTTGGTGGAGTTGGATGTGAGCTTCAACAGT 960

************************************************************

AM_cla011257 CTAACGTACTTGCCAACAAATATTGGCCATGAACTGGTGAATTTGGAGAAGCTTGCTATC 1020

TS_cla011257 CTAACGTACTTGCCAACAAATATTGGCCATGAACTGGTGAATTTGGAGAAGCTTGCTATC 1020

************************************************************

AM_cla011257 CAGTTAAACAAGATACGCTCACTTCCCTCTTCTCTTTGTGGTATGAGCTCTTTGCGTTAT 1080

TS_cla011257 CAGTTAAACAAGATACGCTCACTTCCCTCTTCTCTTTGTGGTATGAGCTCTTTGCGTTAT 1080

************************************************************

AM_cla011257 CTGGATGCGCATTTCAACGAGCTTCATGGCCTTCCTCAGGCAATTGGGAAACTGACACAA 1140

TS_cla011257 CTGGATGCGCATTTCAACGAGCTTCATGGCCTTCCTCAGGCAATTGGGAAACTGACACAA 1140

************************************************************

AM_cla011257 CTTGAGTATCTCAACCTAGGCAGTAATTTTACTGACCTTACTGAACTTCCACATACTTTT 1200

TS_cla011257 CTTGAGTATCTCAACCTAGGCAGTAATTTTACTGACCTTACTGAACTTCCACATACTTTT 1200

************************************************************

AM_cla011257 GGTGATTTATCCAGCCTTAAGGAACTTGATCTCAGCAATAACCAGATTCACGCTCTACC**T** 1260

TS_cla011257 GGTGATTTATCCAGCCTTAAGGAACTTGATCTCAGCAATAACCAGATTCACGCTCTACC**C** 1260

***********************************************************

AM_cla011257 GATACATTTGGCCATCTTGAGAATTTAAAGAAACTAAACTTGGAGCAAAATCCTCTTACA 1320

TS_cla011257 GATACATTTGGCCATCTTGAGAATTTAAAGAAACTAAACTTGGAGCAAAATCCTCTTACA 1320

************************************************************

AM_cla011257 ATTCCACCAATGGAAGTGGTTAACAAAGGACCGGATGCTGTGAGGACATTCATGTCCAAA 1380

TS_cla011257 ATTCCACCAATGGAAGTGGTTAACAAAGGACCGGATGCTGTGAGGACATTCATGTCCAAA 1380

************************************************************

AM_cla011257 AGATGGCTTGAAATTCTGGAAGAGGAAGATAGAAAAAGAACTCTTGAAATGGATGAACAG 1440

TS_cla011257 AGATGGCTTGAAATTCTGGAAGAGGAAGATAGAAAAAGAACTCTTGAAATGGATGAACAG 1440

************************************************************

AM_cla011257 ACGCAGACTGGATGGCTGACACGAAGCACCTCTTGGTTGAAGACTTACGTTTCTGGCGTT 1500

TS_cla011257 ACGCAGACTGGATGGCTGACACGAAGCACCTCTTGGTTGAAGACTTACGTTTCTGGCGTT 1500

************************************************************

AM_cla011257 TCAGAGACGGTGTCTGGTATCGT**A**GGATCTCCCAAATCGCCAAGAGACCCGTATCT**C**GAT 1560

TS_cla011257 TCAGAGACGGTGTCTGGTATCGT**T**GGATCTCCCAAATCTCCAAGAGACCCGTATCT**T**GAT 1560

*********************** ************** ***************** ***

AM_cla011257 CAACAGCTATAA 1572

TS_cla011257 CAACAGCTATAA 1572

************

**<SUN protein sequence_Cla011257_tomato protein 제외>**

protein IQ-DOMAIN 14-like

IQ calmodulin-binding motif (melon의 protein sequence도 포함)

AM_chr3_cla011257 ------------------------------------------------------------

TS_chr3_cla011257 ------------------------------------------------------------

MELO3C015418P1 MGKATRWFKSLFGIKRDKEPTKEIPKSKPPTTAVDVQLCNNPATIPPNLSAAEAAWLKSF 60

AM_chr3_cla011257 ----------------------------------------------MFGGGRERWASVKI 14

TS_chr3_cla011257 ----------------------------------------------MFGGGRERWASVKI 14

MELO3C015418P1 YSETEKEQSKHAIAVAAATAAAADAAVAAAQAAVAVVRLTSHGRGTMFGGGRERWASVKI 120

**************

AM_chr3_cla011257 QTCFRGYLARKALRALKGLVKLQALVRGYLVRKQATATLHSMQALIRAQATVRSQRTRRF 74

TS_chr3_cla011257 QTCFRGYLARKALRALKGLVKLQALVRGYLVRKQATATLHSMQALIRAQATVRSQRTRRF 74

MELO3C015418P1 QTCFRGYLARKALRALKGLVKLQALVRGYLVRKQATATLHSMQALIRAQATVRSQRTRRF 180

************************************************************

AM_chr3_cla011257 INDETRLEIRARKSMERFDDTKSEHTASVHSRRLSASLDNTAFTAMEESPKIVEIDTGRP 134

TS_chr3_cla011257 INDETRLEIRARKSMERFDDTKSEHTASVHSRRLSASLDNTAFTAMEESPKIVEINTGRP 134

MELO3C015418P1 INDS-------RKSTERFEDTKSEHTVSVHSRRLSASLDNTTF--MEESPKIVEIDTGRP 231

***. *** ***:*******.**************:* **********:****

AM_chr3_cla011257 KSWSRRTNTSASELSDDLFHQTLSSPLPCRTPSRLQIPDCRNFHD-SDWCGGDDWRLIST 193

TS_chr3_cla011257 KSWSRRTNTSASELSDDLFHQTLSSPLPCRTPSRLQIPDCRNFHD-SDWCGGDDWRLIST 193

MELO3C015418P1 KSWSRRTNTSASELSDDPFNQTLSSPLPCRTPSRLQIPDCRHFHENSDFCGGDDWRLIST 291

***************** *:*********************:**: **:***********

AM_chr3_cla011257 AQSTPRFVNSGGGSNGPPTPAKSMCGENFFKGYLNFPNYMANTQSFKAKLRSQSAPKQRP 253

TS_chr3_cla011257 AQSTPRFVNSGGGSNGPPTPAKSMCGENFFKGYLNFPNYMANTQSFKAKLRSQSAPKQRP 253

MELO3C015418P1 AQSTPRFMGSGGGSNGPPTPAKSMCGENFFRGYLNFPNYMANTQSFKAKLRSQSAPKQRP 351

*******:.*********************:*****************************

AM_chr3_cla011257 ELGSKKRVSLNELMESRSSLSGVRMQRSCSQVQEAINFKNAVMSKLDRPSEFNNLQRRI 312

TS_chr3_cla011257 ELGSKKRVSLNKLMESRSSLSGVRMQRSCSQVQEAINFKNAVMSKLDRPSEFNNLQRRI 312

MELO3C015418P1 EIGSKKRVSLNELMESRNSLSGVKMQRSCSQVQEAINFKNAVMNKLDRPSEFNNLQRKL 410

*:*********:*****.*****:*******************.*************::

**<SUN protein sequence_Cla011257_tomato protein 포함>**

protein IQ-DOMAIN 14-like

IQ calmodulin-binding motif (melon의 protein sequence도 포함)

AM_chr3_cla011257 ------------------------------------------------------------

TS_chr3_cla011257 ------------------------------------------------------------

MELO3C015418P1 MGKATRWFKSLFGIKRDKEPTKEIPKSKPPTTAVDVQLCNNPATIPPNLSAAEAAWLKSF 60

Solyc06g066430.2.1 -MKKDKENVDNMSNSSDKRDKKRWSFGKSSKESIGVG--DNPVNFPGGVPAVDSNWLRSY 57

AM_chr3_cla011257 ----------------------------------------------MFGGGRERWASVKI 14

TS_chr3_cla011257 ----------------------------------------------MFGGGRERWASVKI 14

MELO3C015418P1 YSETEKEQSKHAIAVAAATAAAADAAVAAAQAAVAVVRLTSHGRGTMFGGGRERWASVKI 120

Solyc06g066430.2.1 ISENEKEQSKHAIAVAAATAAAADAAVAAAQAAVAVVRLTSQGRGAMFTGGREKWAAAKI 117

** ****:**:.**

AM_chr3_cla011257 QTCFRGYLARKALRALKGLVKLQALVRGYLVRKQATATLHSMQALIRAQATVRSQRTRRF 74

TS_chr3_cla011257 QTCFRGYLARKALRALKGLVKLQALVRGYLVRKQATATLHSMQALIRAQATVRSQRTRRF 74

MELO3C015418P1 QTCFRGYLARKALRALKGLVKLQALVRGYLVRKQATATLHSMQALIRAQATVRSQRTRRF 180

Solyc06g066430.2.1 QTVFRGYLARKALRALKGLVKLQALVRGYLVRKRAAATLHSMQALIRAQAAVRSQRARRS 177

** ******************************:*:**************:*****:**

AM_chr3_cla011257 INDETR--LEIRARKSMERFDDTKSEHTASVHSRRLSASLDNTAFTAMEESPKIVEIDTG 132

TS_chr3_cla011257 INDETR--LEIRARKSMERFDDTKSEHTASVHSRRLSASLDNTAFTAMEESPKIVEINTG 132

MELO3C015418P1 INDS---------RKSTERFEDTKSEHTVSVHSRRLSASLDNTTF--MEESPKIVEIDTG 229

Solyc06g066430.2.1 MTNDTRNQPETRARRSIERFDEYRSEF----HSKRLSTS-NDTSYDGFDESPKIVEIDTF 232

:.:. *:* ***:: :**. **:***:* ::*:: ::********:*

AM_chr3_cla011257 RPKSWSRRTN-TSASELSDDLFHQTLSSPLPCRTPSRLQIPDCRNFHD-SDWCGGDDWRL 190

TS_chr3_cla011257 RPKSWSRRTN-TSASELSDDLFHQTLSSPLPCRTPSRLQIPDCRNFHD-SDWCGGDDWRL 190

MELO3C015418P1 RPKSWSRRTN-TSASELSDDPFNQTLSSPLPCRTPSRLQIPDCRHFHENSDFCGGDDWRL 288

Solyc06g066430.2.1 RTKSRSRRMNNACMSESGDEQHSQAMSSPLPCPLPARLSIPDCRHLQDVNWSFLADEQCK 292

*.** *** * :. ** .*: . *::****** *:**.*****:::: . .*:

AM_chr3_cla011257 ISTAQSTPRFVNSGGGSNGPPTPAKSMCGENFFKGYLNFPNYMANTQSFKAKLRSQSAPK 250

TS_chr3_cla011257 ISTAQSTPRFVNSGGGSNGPPTPAKSMCGENFFKGYLNFPNYMANTQSFKAKLRSQSAPK 250

MELO3C015418P1 ISTAQSTPRFMGSGGGSNGPPTPAKSMCGENFFRGYLNFPNYMANTQSFKAKLRSQSAPK 348

Solyc06g066430.2.1 FASAQTTPRFAGSGR-SNAPPTPAKSICGDGYFRAYANFPNYMSNTQSFRAKLRSHSAPK 351

:::**:**** .** **.*******:**:.:*:.* ******:*****:*****:****

AM_chr3_cla011257 QRPELGSKKRVSLNELMESRSSLSGVRMQRSCSQVQEAINFKNAVMSKLDRPSEFNNLQR 310

TS_chr3_cla011257 QRPELGSKKRVSLNKLMESRSSLSGVRMQRSCSQVQEAINFKNAVMSKLDRPSEFNNLQR 310

MELO3C015418P1 QRPEIGSKKRVSLNELMESRNSLSGVKMQRSCSQVQEAINFKNAVMNKLDRPSEFNNLQR 408

Solyc06g066430.2.1 QRPEPGPKKRLSLNEIMASRTSFSGVRMQKSCSQVQEEYCF------------------- 392

**** *.***:***::* **.*:***:**:******* *

AM_chr3_cla011257 RI 312

TS_chr3_cla011257 RI 312

MELO3C015418P1 KL 410

Solyc06g066430.2.1 --
